# Polymeric Sorbents as Energy‐Efficient Alternative to Cryogenic Distillation for Light Hydrocarbon Purification

**DOI:** 10.1002/adma.74471

**Published:** 2026-08-10

**Authors:** Kelechi Festus, Ankit Mondal, Suman Mandal, Jazmine Aiya D. Marquez, Arnab Ghosh, Aishi Sikdar, Urme Podder, Ahana Sarkar, Qingsheng Wang, Hong‐Cai Zhou

**Affiliations:** ^1^ Department of Chemistry Texas A&M University College Station Texas USA; ^2^ Artie McFerrin Department of Chemical Engineering Texas A&M University College Station Texas USA

**Keywords:** cryogenic distillation, energy, light hydrocarbons, porous materials, purification, separation

## Abstract

Light hydrocarbons are essential gaseous compounds for fuel, polymer manufacturing, fertilizer production, and so forth. The efficient recovery and purification of these gases are still challenging despite their significant industrial and domestic relevance. They exist in the gaseous state under ambient temperature and pressure and are typically recovered from natural gas streams containing multiple close‐boiling‐point hydrocarbons. So far, cryogenic distillation is the commonly used method for their separation and purification. It operates based on their boiling‐point differences, which amounts to significant energy consumption. To address this limitation, adsorption‐based technology is considered an energy‐efficient alternative for the purification of light hydrocarbons. To this effect, polymeric sorbents such as porous polymer networks (PPNs) are a potential alternative due to their robustness, stability, and scalability. This review provides an extensive discussion on cryogenic distillation, applications of light hydrocarbons, and progress in light hydrocarbon purification using polymeric sorbents. Additionally, the pore structure, stability, and techno–economic feasibility of the polymeric sorbents were extensively examined, covering current progress, mechanism of guest molecule separation, characterization techniques, and hurdles still encountered. Overall, since their application in light hydrocarbon separation is still an evolving field, this review will facilitate robust designs by providing the necessary know‐how and roadmaps needed for advancement.

## Graphical abstract

The transition from cryogenic distillation to polymeric sorbents for light hydrocarbon purification is crucial for energy and environmental sustainability. The polymeric sorbents are engineered to separate gas mixtures based on their specific properties.

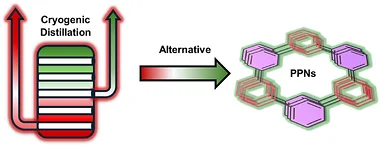

## 1 Introduction

The design and development of alternative solid‐state materials for the purification of light hydrocarbons have drawn significant attention in recent times [1, 2, 3, 4, 5, 6, 7]. This is because of the ever‐growing population of the world, which has led to increased demand for various consumers' high‐end commodities [8]. Natural gas processing to generate light hydrocarbons is a vital source of energy and raw materials for the manufacturing of various products whose demand has necessitated the development of modern and efficient technology for their harvesting and purification. This is because they are usually contaminated with unwanted moieties. Currently, cryogenic distillation is the established method for purifying them with a significant energy penalty, which accounts for ca. 0.3% of global energy consumption [9, 10]. As a result, this renders its continuous use unfeasible in the long run, and hence the need for an alternative that is robust, efficient, reliable, and techno‐economically feasible [11, 12]. The mitigation of this challenge to promote the continuous availability of raw materials has drawn the interest of industries, governments, and non‐government organizations globally and has also provided a new roadmap and milestones for researchers to explore.

Specifically, cryogenic distillation has gained popularity and significance due to its ability to separate lightweight or short‐chain hydrocarbons based on their boiling points. It is a large‐scale industrial process that requires powerful distillation towers to achieve the separation of these gases. Short‐chain hydrocarbons have been purified through this technique for quite a long time, and gases such as ethylene and methane are very crucial to industries because of the purpose that they serve. For example, ethylene is used as a starting material for polymerization to produce polymer‐based products for vehicle parts, electronic appliances, and domestic use [13]. As a byproduct of natural gas processing, ethylene is contaminated with ethane, while methane, a source of energy, is contaminated with propane and ethane, which decreases ignition time and makes them dangerous in the crude state [7, 14]. The removal of these impurities has been done with cryogenic distillation in the petrochemical industries for a long time, relying heavily on the close range of their boiling points [15]. Although this technique has proven to be very efficient over time and finds diverse applications in the industry, its run‐time cost, hazards, design, and development expenditure cannot be sustained, which has necessitated the quest for a more facile and robust alternative [16, 17, 18, 19, 20].

PPNs have been actively developed as a potential alternative to cryogenic distillation because their pores can be engineered to fit the kinetic diameter of target gases for efficient purification from contaminants [4, 7, 21, 22]. They are solid‐state materials that possess ultrahigh thermal and chemical stability and hence can be applied in harsh environments. Several sorbents have been investigated for the separation of light hydrocarbons such as C_2_H_6_ and C_2_H_4_ in binary mixtures and C_3_H_8_, C_2_H_6_, and CH_4_ in ternary mixtures, and these include metal‐organic frameworks (MOFs) [23, 24, 25, 26, 27, 28, 29, 30, 31, 32, 33], porous polymer networks (PPNs) [7, 14, 15, 34, 35, 36], covalent organic frameworks (COFs) [2, 37, 38, 39, 40, 41, 42], and zeolites [6, 43, 44, 45, 46]. Amongst all, PPNs are more robust and promising for commercialization and application in the industry because their building blocks (monomers) are cheap and readily available. The synthesis of PPNs with specific properties, such as thermal and chemical stability, techno‐economic feasibility, and low regeneration energy, is facile and unambiguous. Additionally, PPNs’ structural integrity is not compromised after multiple regeneration cycles because they are amorphous and metal‐free. Although a myriad of sorbents have been reported in the literature, many of them still suffer from weak adsorption kinetics, poor adsorption and separation potential, strong guest to sorbent interaction requiring high regeneration energy, and inability to withstand industry standard multiple regeneration cycles, which necessitates intensified design strategy and optimization [47, 48]. These will lead to efficiency in gas separation, availability of raw materials for industrial processes, reduced cost of living, and replacement of cryogenic distillation.

In this review, we examined the strategy that has been adopted for hydrocarbon separation over the years, specifically cryogenic distillation. Just as important, we also discussed the importance of light hydrocarbons and explored PPNs as an alternative to cryogenic distillation. Mechanisms of gas separation and relevant characterization techniques applicable in PPNs synthesis and their structure‐property elucidation were also buttressed to facilitate the advancements of PPNs as robust and promising alternatives to cryogenic distillation technology.

## 2 Light Hydrocarbons and Their Applications

Light hydrocarbons (LHs) are petroleum‐derived intermediate products in the catalytic decomposition of oil to gas [49]. Generally, these intermediates are hydrocarbon compounds composed of hydrocarbons containing 1–13 carbon atoms [50]. The wide range of carbon content further classifies LH into two phases at ambient conditions: vapor‐phase hydrocarbons and liquid‐phase hydrocarbons [51]. The former is made up of short‐chain hydrocarbons with 1–5 carbon content. These are small molecules with low molecular weight and surface area, as well as weak intermolecular forces that can easily be overcome, allowing them to exist in the gaseous phase at ambient conditions. On the other hand, the latter is composed of 6–13 carbon atoms in its chain. The increased number of carbons in the chain also increases molecular weight and surface area, which increases intermolecular interaction. This leads to higher energy needed for the long‐chain hydrocarbon to be in the gaseous phase in ambient conditions, hence the liquid‐phase. Both types of LHs are used as energy sources, as well as chemical feedstock to produce everyday commodities. Of the two types, the liquid‐phase hydrocarbons mixed with C_5_ and C_4_, also known as linear α‐olefins, are more valuable because of their high reactivity, economic efficiency, and high‐value‐added product derivatives [52]. On the other hand, the abundance of C_1_–C_3_ because of the discovery of shale gas (as shown in Figure 1) allowed natural gas to become cheaper [53]. Hence, there is a demand to utilize these products further as an energy source and a feedstock for important chemicals.

**FIGURE 1:**
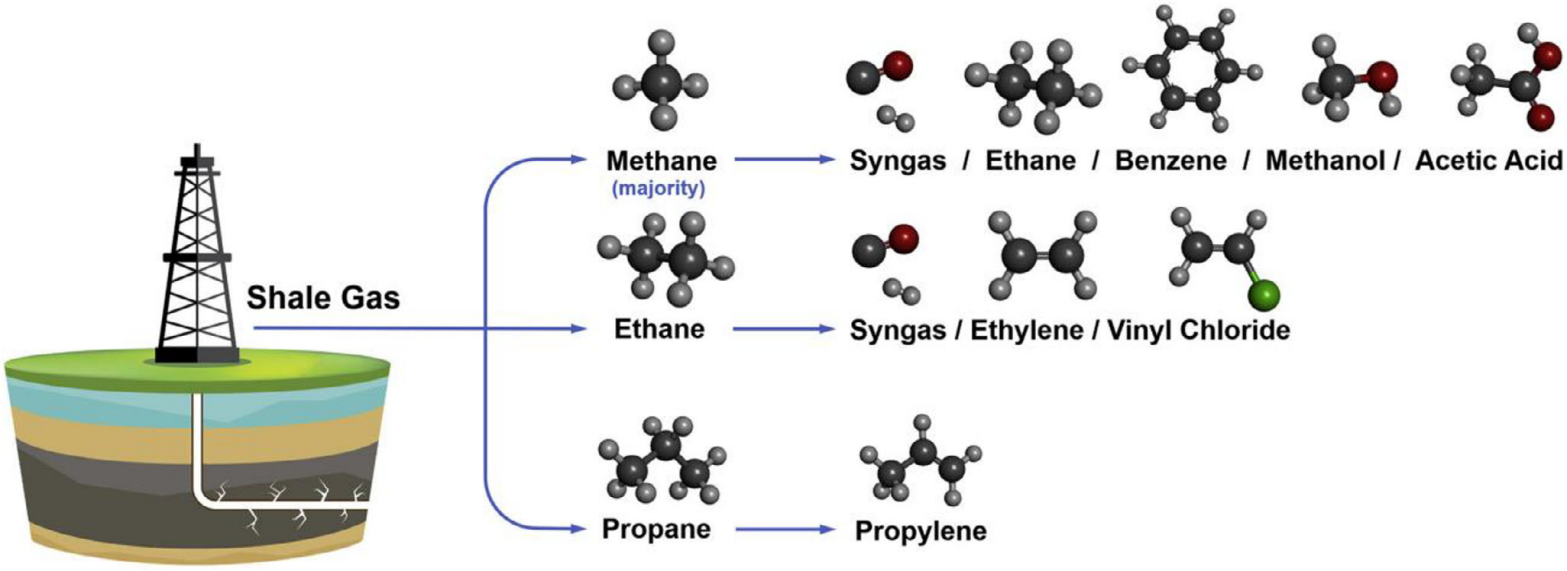
Schematic representation of shale gas as a source of light hydrocarbons (methane, ethane, and propane), which are essential feedstock for various high‐value chemicals. Reproduced with permission [54]. Copyright 2021, Elsevier.

### 2.1 Linear α‐Olefin (LAO): C_4_–C_13_ Light Hydrocarbons

LAO is an important mixture found in crude oils that makes up 90% of light oil or condensates after fractional distillation [55]. In this context, several studies have shown the possible applications of LAOs. Ghannam, Hasan [56] investigated the effects of LAO containing C_5_–C_13_ hydrocarbons on the flowability of heavy crude oil and showed that light oils can eliminate the thixotropic behavior of heavy crude oil, as well as diminish apparent yield stress. The reviews of Golub et al. emphasized the importance of different processing techniques for processing LAOs because of their use to produce consumer goods, such as plasticizers, synthetic lubricants, surfactants, co‐polymers, and sulfonates [52, 57]. Of the different hydrocarbons that compose LAOs, C_4_ is a four‐carbon vapor‐phase LH that is colorless and easily liquifiable. It is a valuable product that is often separated from higher carbon chains and utilized independently. It is used for domestic purposes, subcritical extractions, refrigerants, and raw material synthesis [58]. Moreover, LAOs are normally utilized as a mixture of different hydrocarbon chain lengths, commercially synthesized through the oligomerization of ethylene and n‐alkanes using catalysts [52].

### 2.2 Methane: C_1_ Light Hydrocarbon

Methane (C_1_) is a single‐carbon vapor‐phase LH that is colorless, odorless, and a major component of natural gas deposits. It is used primarily as a fuel source that burns cleaner and is more efficient than long‐chain hydrocarbons [59]. However, it is also a known anthropogenic greenhouse gas with a global warming potential that is about 28 to 48 times higher than CO_2_ [60, 61]. Nonetheless, its abundance and its ability to be recovered categorize it as an abundant energy resource, which has attracted researchers to probe other crucial products generated from it (Figure 2a) and its application as source heat (Figure 2b) [62]. An example is traditional energy generation through the combustion of methane, which could be improved through catalytic combustion that increases methane utilization efficiency and reduces other pollutants such as NO_x_ [63]. On the other hand, Li et al. have examined the feasibility of using chemical‐looping conversion of methane, which showed that it is a versatile technique with the ability to achieve energy generation, CO_2_ capture, and pure syngas production through the reaction of methane with different oxygen carriers [64]. It is also worth noting that methane utilization is not limited to direct energy generation. It can also be used as a feedstock for other valuable commodities. For instance, in solar energy, the introduction of partial methane oxidation improves the efficiency of solar cell energy production. Ruan et al. presented the improved efficiency of solar thermochemical CO_2_ splitting and solar thermochemical water splitting processes through the integration of methane‐driven reduction that utilizes partial oxidation of methane [65]. Similarly, a cyclic redox scheme was proven to enhance CO_2_‐splitting through the partial oxidation of methane that promotes the reduction by iron‐containing mixed‐oxide nanocomposites by Li et al. [66]. Furthermore, the manufacture of methanol, ethylene, aromatics, and hydrogen, which are essential commodities, is done with methane [67]. Srivastava et al. discussed several multidisciplinary approaches to convert methane to methanol that minimize the adverse environmental effects of methane, such as supercritical water, photocatalysts, and plasma technology [68]. In contrast, oxidative and nonoxidative methane conversion processes have been proven to be effective in producing ethylene [69], while nonoxidative methane dehydroaromatization has successfully been used for hydrogen and benzene production [70]. However, the main drawback of these processes is catalyst efficiency, high energy intensity, and methane purity.

**FIGURE 2:**
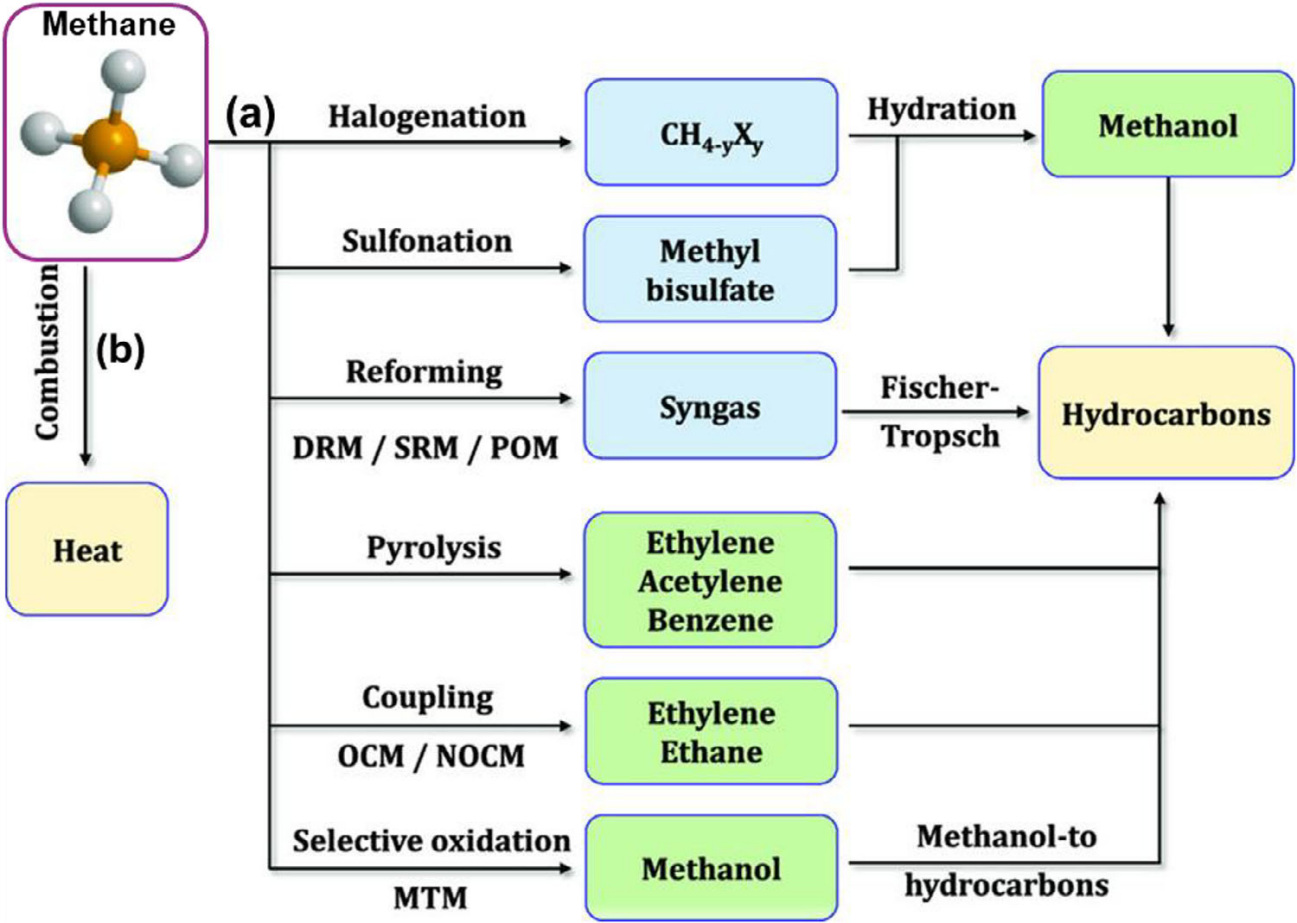
Schematic illustration of methane as a feedstock. (a) Methane transformation pathways into value‐added products. (b) Combustion of methane for energy and heat generation [62]. This demonstrates the various channels through which methane can be transformed into useful moieties and heat. Copyright 2019, Wiley‐VCH.

### 2.3 Ethane and Ethylene: C_2_ Light Hydrocarbon

Ethane (C_2_) is a two‐carbon‐length vapor‐phase LH that is the second largest component of natural gas and shale gas, and can be obtained in abundance in shale gas fields [71]. Consequently, this surplus has facilitated a drop in ethane price, boosted its availability, and promoted its use as a primary feedstock for chemical production, which is traditionally obtained from naphtha [72]. Additionally, it is widely used as a feedstock for plastics manufacturing [73]. Other applications include the regeneration of used lubricant oil through ethane‐based extraction [74], ethylene production by partial oxidation of ethane [75], generation of aromatic hydrocarbons from direct aromatization of ethane using catalysis [71, 76]. However, one current challenge in the industry is separating excess ethane from desired products after it has been processed, especially ethane and ethylene [9]. The purification process is energy‐intensive and laborious, which remains a major problem in the chemical and manufacturing industry [77].

Ethylene (C_2_) plays a major role in the industry as a crucial building block for many chemical and petrochemical products [78]. Although it is a two‐carbon‐length vapor‐phase LH, it is made up of a C═C (double bond). It is produced from ethane and naphtha steam cracking, and several products can be obtained from its processing or upcycling (Figure 3) [69, 79]. It is widely used in the plastics industry as the building block of both commodity and engineering plastics [80], such as polyethylene. It can also be converted to other high‐value products, such as ethylene glycol [81] and ethylene oxide [82, 83], which are later used to synthesize polymers such as polyethylene glycol [84], polyethylene terephthalate [85], and polyethylene oxide [86]. It is also a raw material for the production of other valuable commercial products, such as polyvinyl chloride, adhesives, fibers, and fumigants [79]. In these applications, the purity of ethylene is crucial because it can affect polymer molecular weight, structure, and uniformity [87].

**FIGURE 3:**
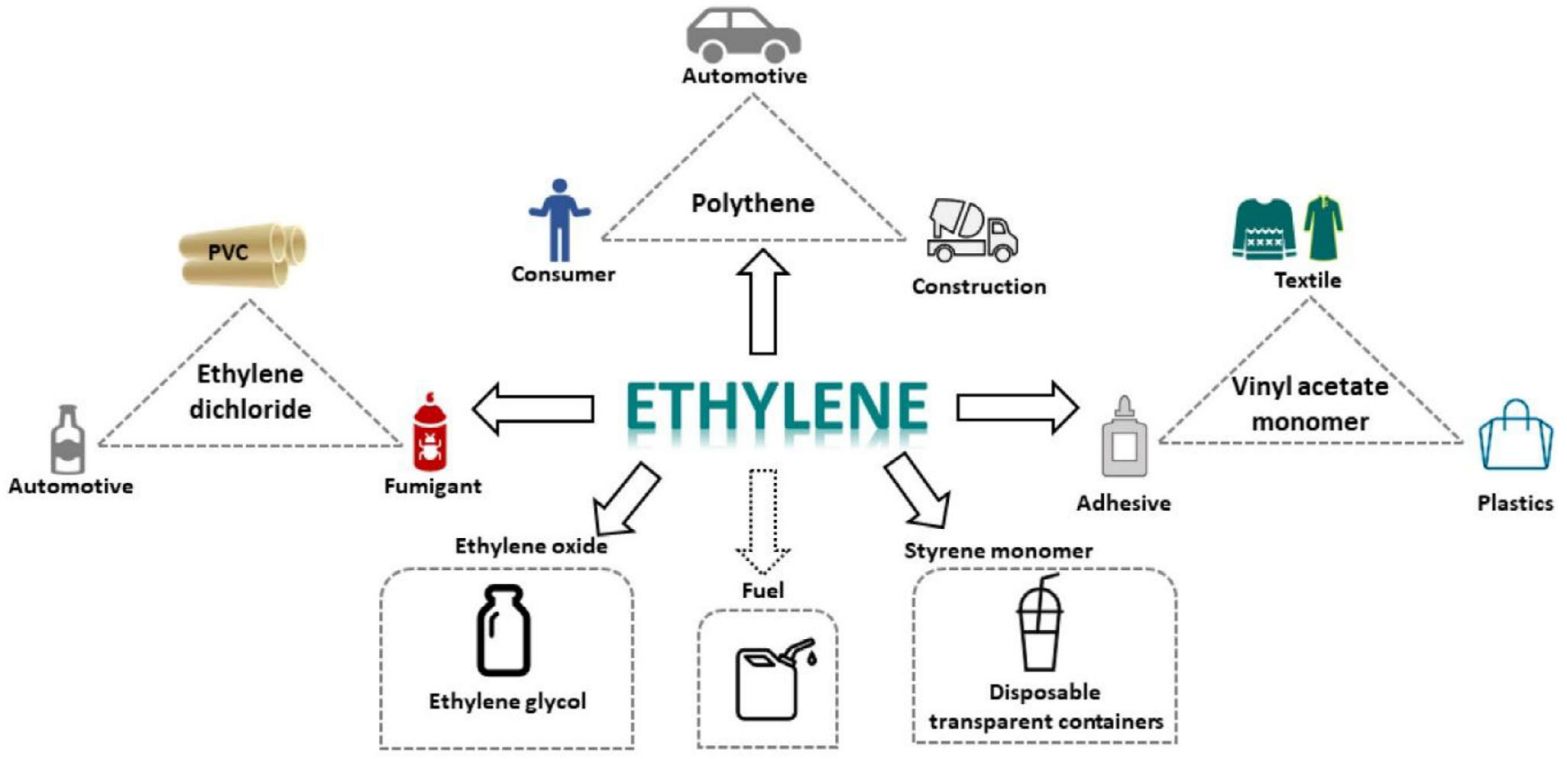
Applications of products obtained from ethylene. Reproduced with permission [79]. Copyright 2023, American Chemical Society.

### 2.4 Propane: C_3_ Light Hydrocarbon

Propane (C_3_) is a three‐carbon‐length vapor‐phase LH that is a byproduct of natural gas and crude oil processing [88, 89, 90]. It is retailed for everyday use in its liquid form, known as liquefied petroleum gas (LPG). Typically, LPG is used as fuel for light‐, medium‐, and heavy‐duty propane vehicles [91], as well as household stoves, burners, and heaters [92]. In the industry, propane is used as a raw material for the production of various chemicals. The most notable ones are the manufacture of propylene from propane, illustrated by Ding et al. (Figure 4) [93]. These derivatives are important feedstocks necessary for different plastics and industrial products and are developed through catalytic propane dehydrogenation. Other industrial applications of propane include: the regeneration of used lubricant oil [94], and a solvent for heavy oil dewaxing, deasphalting, and refining [95]. Although propane has been a general commodity for both domestic and industrial use, its source has been considered limited. Today, propane generated from hydro‐processed esters and fatty acids (HEFA), also called “green propane”, is being explored as a renewable source. However, its scale‐up feasibility is limited by the purification step of propane because it increases capital investments by 6.5%–8.5%, which is crucial for small‐scale plants [96].

**FIGURE 4:**
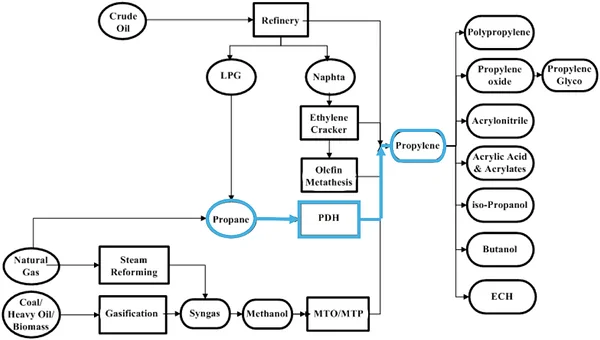
Value chain of propylene that shows propane as an avenue of propylene production (shown in light blue) through propane dehydrogenation (PDH). Reproduced with permission [93]. Copyright 2012, Wiley‐VCH.

Overall, LHs are important raw materials in the energy, industrial, and chemical sectors. With the demand for sustainable alternatives to fossil fuel‐derived LHs, new sources of these compounds are bound to be discovered and developed. Hence, it is imperative to have a purification system that is economically feasible, environmentally friendly, and future‐ready. PPN exploration and development for LH purification is necessary as an alternative to cryogenic distillation to make this essential feedstock readily available.

## 3 Light Hydrocarbon Separation Using Cryogenic Distillation

Separations of light hydrocarbons are especially challenging due to their small molecular‑size differences and closely spaced boiling points, which result in inherently low driving forces for separation (Figure 5) [97, 98]. Cryogenic distillation therefore exploits low‑temperature thermodynamics to access vapor–liquid equilibrium (VLE) regimes where volatility differences become adequate for practical separation [97, 99, 100]. The thermodynamic foundation for demethanizer and deethanizer design is established by extensive experimental VLE data for methane–ethane–propane and methane–propane–n‑butane systems at low temperatures and high pressures, which show that relative volatilities rise significantly under cryogenic conditions [101, 102, 103]. Empirical equations of state, such as the Benedict–Webb–Rubin formulation, are used to represent such phase‑equilibrium behavior and remain widely applied for modeling light‑hydrocarbon mixtures in cryogenic separation processes [104]. These thermodynamic foundations underpin steady‑state simulation and design of cryogenic distillation columns used in industrial natural gas liquids (NGL) recovery systems [105].

**FIGURE 5:**
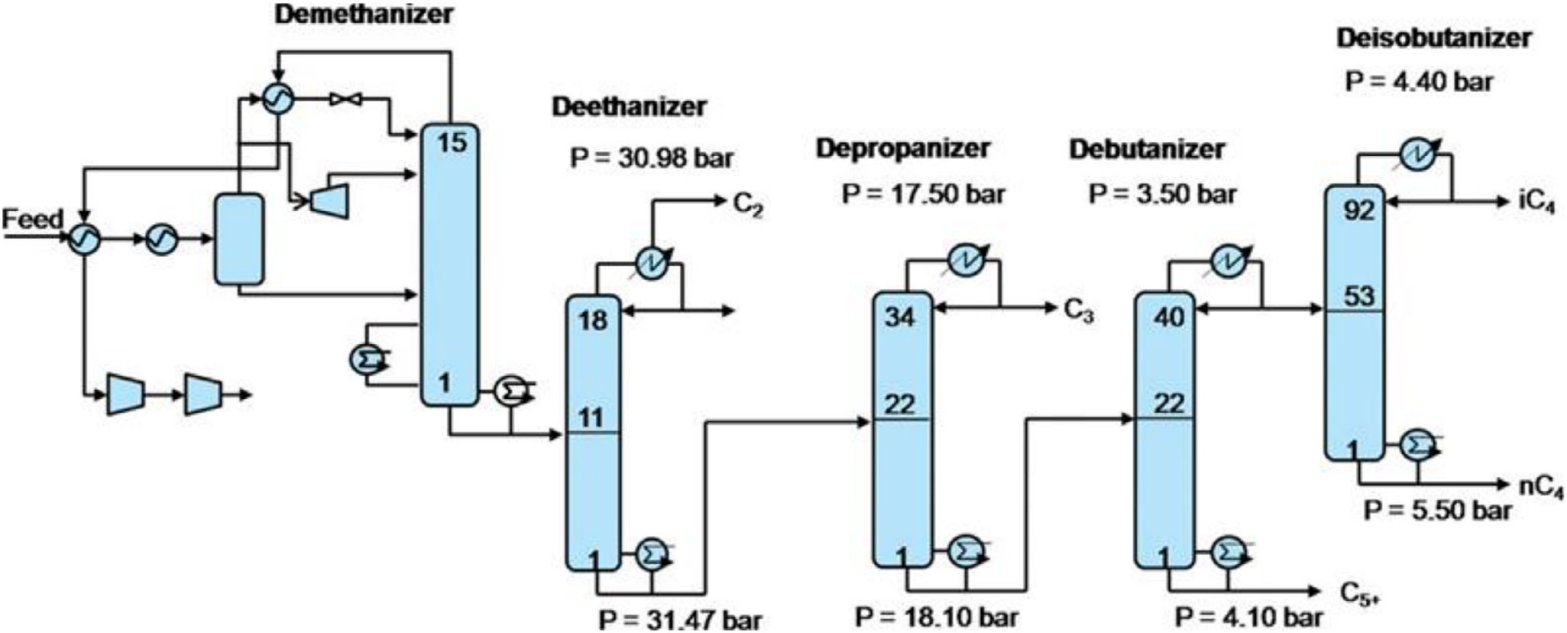
Schematic representation of a conventional natural gas liquids (NGLs) fractionation train, in which sequential cryogenic distillation columns (deethanizer, depropanizer, debutanizer, and optional butane splitter) separate light hydrocarbons from C_2_ to C_5_^+^ based on increasing boiling points. Reproduced with permission [98]. Copyright 2012, American Chemical Society.

In light hydrocarbons separation via cryogenic distillation, separation is achieved through a sequence of distillation columns [99]. A conventional NGL fractionation train sequentially consists of a demethanizer, deethanizer, depropanizer, and debutanizer (Figure 5) [98, 106]. Methane is the first removed from the feed mixture in a very low temperature distillation step, and the liquid C_2_+ stream is processed downstream. In the next step, the ethane‐rich overhead gets separated from the heavier NGL‐mixture, which enters the depropanizer, and the debutanizer afterward, and thus yields butane‐rich liquefied petroleum gas (LPG) overhead and C_5_+ natural gasoline or condensate fraction at the bottom of the column. The demethanizer is the initial column and is considered the key unit governing the performance of the entire separation process. Systematic variation in pressure, reflux, or feed conditions of the demethanizer can significantly alter the composition, temperature, and flow of the NGL feed entering the deethanizer, with direct consequences for energy consumption and product quality in the downstream fractionation columns [107, 108, 109]. Experimental cryogenic distillation data obtained at industry‐relevant conditions (up to 4 MPa and down to 233 K) demonstrate the strong influence of column operating pressure and temperature on phase behavior and separation performance, and provide critical data for improving the prediction and design of LNG cryogenic demethanizer columns [110]. Process‐level optimization and system analysis also confirm that upstream demethanizer conditions propagate through the fractionation train, impacting the overall process efficiency in both conventional NGL and LNG‐integrated systems. For NGL fractionation, optimization of distillation column sequencing, including the demethanizer and downstream columns, demonstrates that column configuration significantly influences energy consumption and process performance [111]. Just as important, LNG regasification optimization studies highlight how configuration choices affect energy use and C_2_+ recovery [112].

Recent studies have explored process modifications and alternative strategies aimed at improving the efficiency of the demethanizer and enhancing the performance of the overall separation process. Using LNG regasification cryogenic energy, Gao et al. demonstrated viable strategies for recovery of light hydrocarbons (C_2_+) from methane‐rich liquefied natural gas [113]. A comparison of high‐pressure (Figure 6a) and low‐pressure demethanizer (Figure 6b) study showed that the cascade utilization of LNG cryogenic energy can either reduce downstream gas compression or enable methane re‐liquefaction prior to pressurization, thereby lowering the overall energy demand. However, high ethane recovery (>85%) is achieved in both processes, with low pressure demethanizer as the more economically favorable option. While LNG‐integrated cryogenic demethanization effectively recovers ethane and LPG using LNG cold energy, conventional NGL recovery is typically achieved through techniques like turboexpander, Joule–Thomson (JT), or hybrid refrigeration‐JT process. Among these, JT processes typically exhibit low to moderate ethane recovery, whereas turboexpander achieves substantially higher ethane recovery (∼75%) and exergy efficiency (∼75%), together with significantly lower specific energy consumption [114].

**FIGURE 6:**
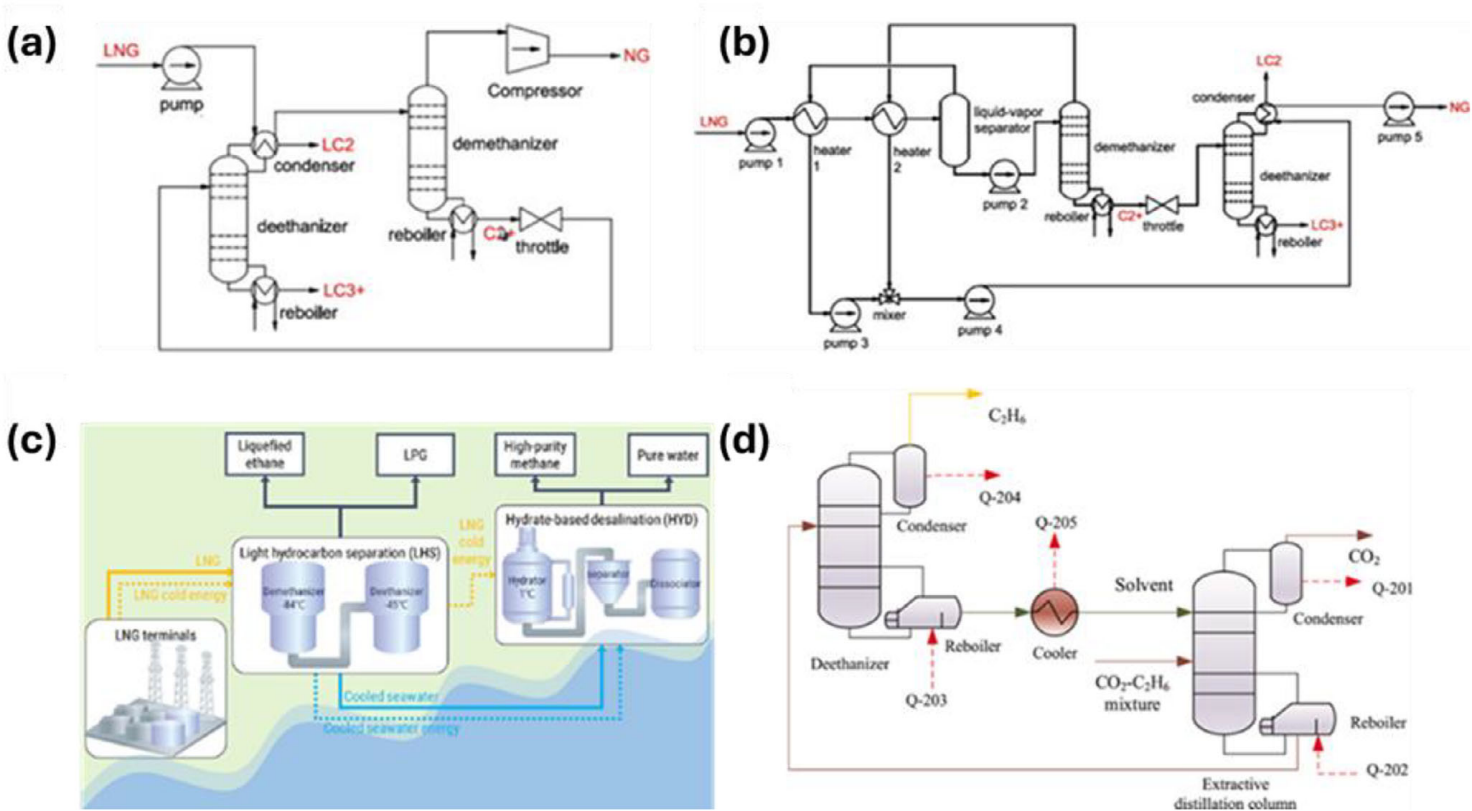
LNG cold‐energy‐assisted cryogenic light hydrocarbon separation schemes employing. (a) a low‐pressure (2.4 MPa) demethanizer enabling cascade cold‐energy utilization and methane re‐liquefaction, and (b) a high‐pressure (4.5 MPa) demethanizer allowing direct compression of methane‐rich natural gas. Reproduced with permission [113]. Copyright 2011, Elsevier. (c) Schematic illustration of an integrated process combining cryogenic light‐hydrocarbon separation with hydrate‐based desalination using LNG cold energy. Reproduced with permission [115]. Copyright 2023, Elsevier. (d) Extractive distillation scheme for separating the CO_2_–C_2_H_6_ azeotropic mixture, in which heavy hydrocarbon solvents enable CO_2_ removal in the extractive column and high‐purity ethane recovery in the downstream deethanizer. Reproduced with permission [116]. Copyright 2024, Elsevier.

Beyond standalone basic turboexpander‐demethanizer configurations, recent studies have increasingly focused on integrating light‐hydrocarbon separation units with other cryogenic processes to improve overall system efficiency while preserving the fundamental principles of cryogenic distillation‐based NGL recovery. Li et al. investigated the integration of LNG cold energy recovery into refinery flowsheets as a substitute for conventional compression‐based refrigeration [117]. This proposed mechanism was shown to recover almost 14 MW of cold energy, resulting in an approximate 8 MW reduction in compression power demand and a cold‐energy utilization efficiency of about 72%, demonstrating effectiveness in the recovery of light (C_2_+) hydrocarbons from methane‐rich refinery gas streams. Beyond conventional light hydrocarbon separation, Park et al. demonstrated a process‐integration strategy in which LNG cold energy is utilized for cryogenic light hydrocarbon separation and hydrate‐based seawater desalination [115]. This study showed that coupling with desalination does not compromise the hydrocarbon separation performance, producing high‐purity methane and liquefied ethane, illustrating the potential of extending overall utilization of LNG‐cold energy (Figure 6c). Zhang et al. investigated the energy integration of LNG‐based light hydrocarbon recovery with cryogenic air separation, demonstrating that LNG cold energy can be effectively utilized to partially offset the refrigeration demand of the air separation unit [118]. Their results showed that the LNG cold‐energy utilization ratio increased by 35.77%, while the deethanizer successfully separated the feed into ethane and LPG, producing a lean natural‐gas stream containing 99.42 mol% methane and a liquefied ethane product with 99.37 mol% purity. Similarly, in another study Pan et al. developed a thermo‐economically optimized LNG energy‐integration framework linking cryogenic light‐hydrocarbon separation, cold‐energy assisted power generation and boil‐off gas combustion, showing that coordinated integration of these units can significantly enhance overall energy efficiency and achieve 81.07% utilization of LNG cold energy alongside improved economic performance [119]. By integrating ethane recovery and cryogenic CO_2_ capture into a dual mixed refrigerant LNG process, He et al. demonstrated high purity ethane recovery and effective CO_2_ capture by exploiting the azeotropic behavior of ethane‐CO_2_ mixture (Figure 6d) [116]. The optimized system showed that for ethane‐rich feeds (2–20 mol%), CO_2_ can be reduced to below 50 ppm while achieving 99.5% ethane recovery rate and purity with a specific power consumption of approximately 0.40 kWh/Nm^3^, offering lower energy consumption than amine‐based chemical adsorption.

Although cryogenic distillation remains as the industrial standard for large scale separation for hydrocarbons, its high energy demand has motivated the development of various energy‐integration strategies or hybrid separation concepts to mitigate the operational cost. The high energy demand of this process lies in the basic thermodynamic principles behind cryogenic separation associated with achieving very low temperatures for effective separation. Studies on a membrane‐distillation hybrid system for C_2_/C_3_ separations show that it can reduce the energy cost by partially replacing the distillation process with membranes [120]. Similarly, seawater can be used as the heat source, which has been shown to achieve high ethane and LPG recovery (above 90%) from LNG, reducing the need for high external energy input [121]. In parallel, alternative strategies based on porous materials such as PPNs [122, 123, 124] have attracted growing interest due to their lower energy requirements and tunable pore structures. This approach demonstrates promising energy efficiency, practical application, scalability, stability, and process integration that are currently being explored.

## 4 Brief Overview of the History of Cryogenic Distillation

The concept of cryogenics came into reality when Guillaume Amontons first proposed the existence of absolute zero [125]. The origins of cryogenic distillation trace back to early efforts to liquefy gases, beginning with the work of Louis Clouet and Gaspard Monge, who reported the first successful liquefaction around 1780 [126]. This was followed by Michael Faraday's experiments in the 1820s and 1840s, which showed that many gases could be turned into liquids by cooling and compressing them. However, some “permanent gases”, like oxygen, nitrogen, methane, and carbon monoxide, did not turn into liquids at that time [126]. These efforts led to the breakthroughs of Cailletet and Pictet in the late 1800s. In 1877, they both independently succeeded in turning nitrogen and oxygen into liquids, which was the first time permanent gases had been successfully turned into liquids [125, 126]. Carl von Linde's continuous refrigeration cycles, which harnessed the Joule–Thomson effect, represented a significant advancement in industrial gas processing. His 1895 air‐liquefaction machine enabled the large‐scale liquefaction of air, paving the way for contemporary cryogenic technology, which was later improved by Georges Claude who added an expansion machine to the liquefaction cycle [127]. Linde and Georges Claude capitalized on this capability, discovering that fractional distillation could effectively separate liquefied air into its constituent components, owing to their varying boiling points [126]. This led to Claude's 1902 air separation plant, which is often thought of as the first commercial cryogenic distillation unit. The development of industrial air separation technologies led to the large‐scale production of oxygen and nitrogen. This was important for steelmaking, chemical processes, and, later, military uses [126, 128, 129].

Apart from air separation, cryogenic distillation has become a crucial tool in the petrochemical industry. It has been used to separate hydrocarbons that have very low and close boiling points, like ethylene/ethane and propylene/propane [130]. Due to such relatively low boiling points, the process has to be done at very low temperatures and often needs a lot of reflux [131, 132]. For reference, a simplified diagram of a cryogenic distillation setup is shown in Figure 7. These industrial uses made cryogenic distillation one of the standard methods for separating light gases on a large scale with high purity for over 60 years [133].

**FIGURE 7:**
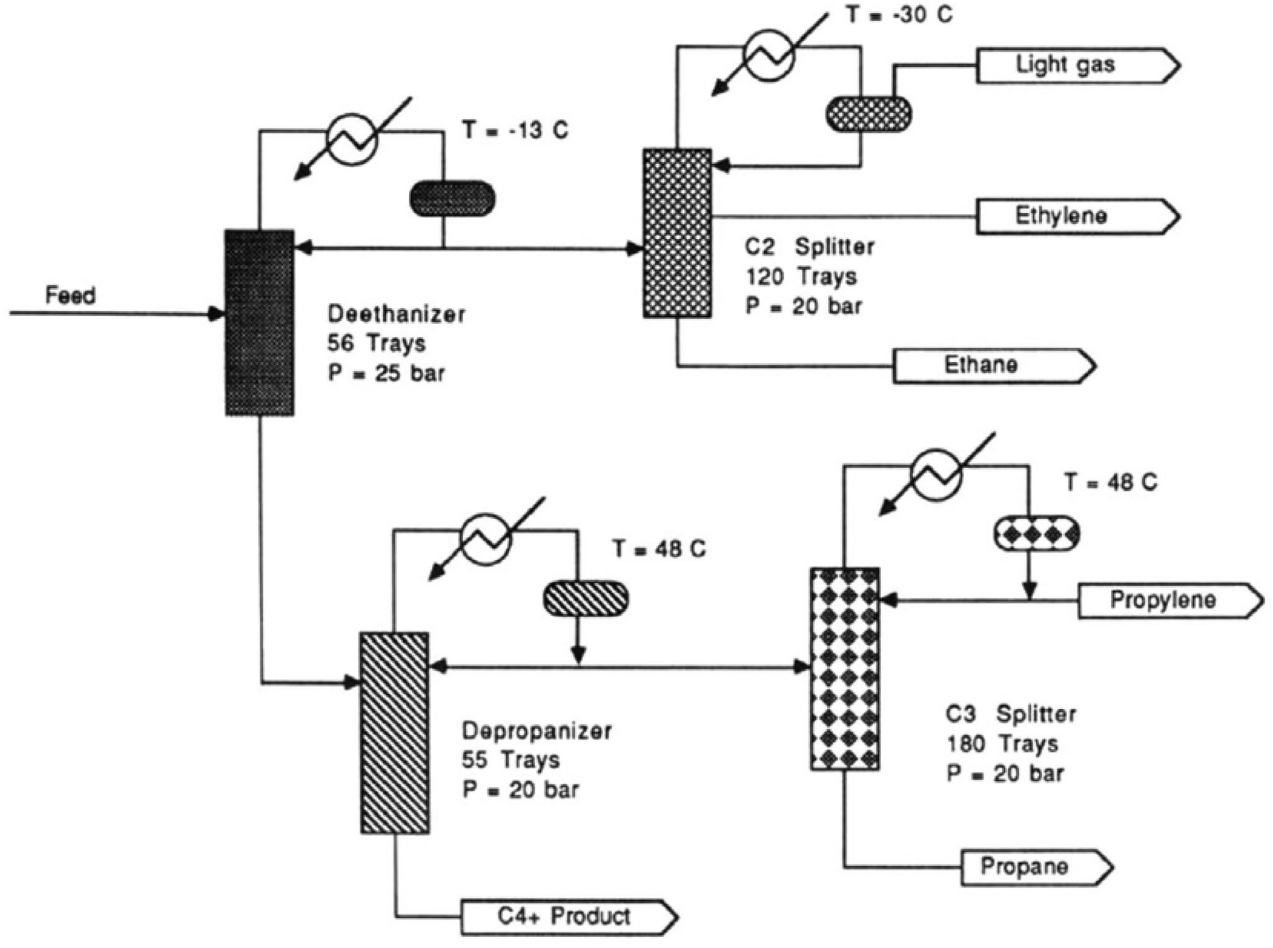
Conventional cryogenic distillation setup for the separation of olefins and paraffins. Reproduced with permission [134]. Copyright 1993, American Chemical Society.

Starting in the 1960s, the technology was gradually improved by better column internals, structured packings, and better heat integration. These advances increased separation efficiency and enabled larger‐scale units [129, 135, 136, 137, 138]. Despite these advancements, the central challenge remains that maintaining cryogenic temperatures requires a lot of energy. Recent studies highlight the need for better strategies, such as heat recuperation and integration techniques, to reduce energy use [139, 140, 141, 142]. As a result, alternative methods like membranes, hybrid systems, and adsorption‐based separations have gained significant attention [143, 144]. PPNs have also emerged as a promising alternative. This is because their permanent porosity, structural and thermal stability, and tunable chemistry allow them to selectively adsorb gas near room temperature [124, 145, 146, 147]. This approach will reduce the energy needed for cryogenic processes in the industries [148].

## 5 Porous Polymer Networks: History, Concept, and Applications

The concept of porosity—the strategic engineering of empty voids within solid matter—has served as a cornerstone of materials science for over a century. These pores are generally interconnected and serve as a medium for the passage of fluids [149]. IUPAC classifies these pores into three categories based on their size: micropores (less than 2 nm), mesopores (between 2 and 50 nm), and macropores (larger than 50 nm) [150, 151]. PPNs are a subset of porous materials that have a hydrocarbon backbone and certain light heteroatoms (B, N, O, etc.) connected by strong covalent bonds [152]. They offer a range of advantages, including light skeletal density, high physicochemical stability, large surface area, and extendibility, which make them suitable for versatile applications such as gas storage/separation, catalysis, sensing, energy storage, and drug delivery, among others [153, 154, 155, 156, 157, 158, 159, 160, 161]. PPNs are further classified into seven categories: hypercrosslinked polymers (HCPs), covalent triazine frameworks (CTFs), porous aromatic frameworks (PAFs), covalent organic frameworks (COFs), polymers of intrinsic microporosity (PIMs), conjugated microporous polymers (CMPs), and porous polymer networks (PPNs). The sequential order of development of these categories of PPNs is summarized in Figure 8, HCP [162], 1969, PIMs [146], 2002, COF (COF‐1) [163], 2005, CMP (CMP‐1) [164], 2007, CTF [165], 2008, and PAF (PAF‐1) 2009 [166].

**FIGURE 8:**
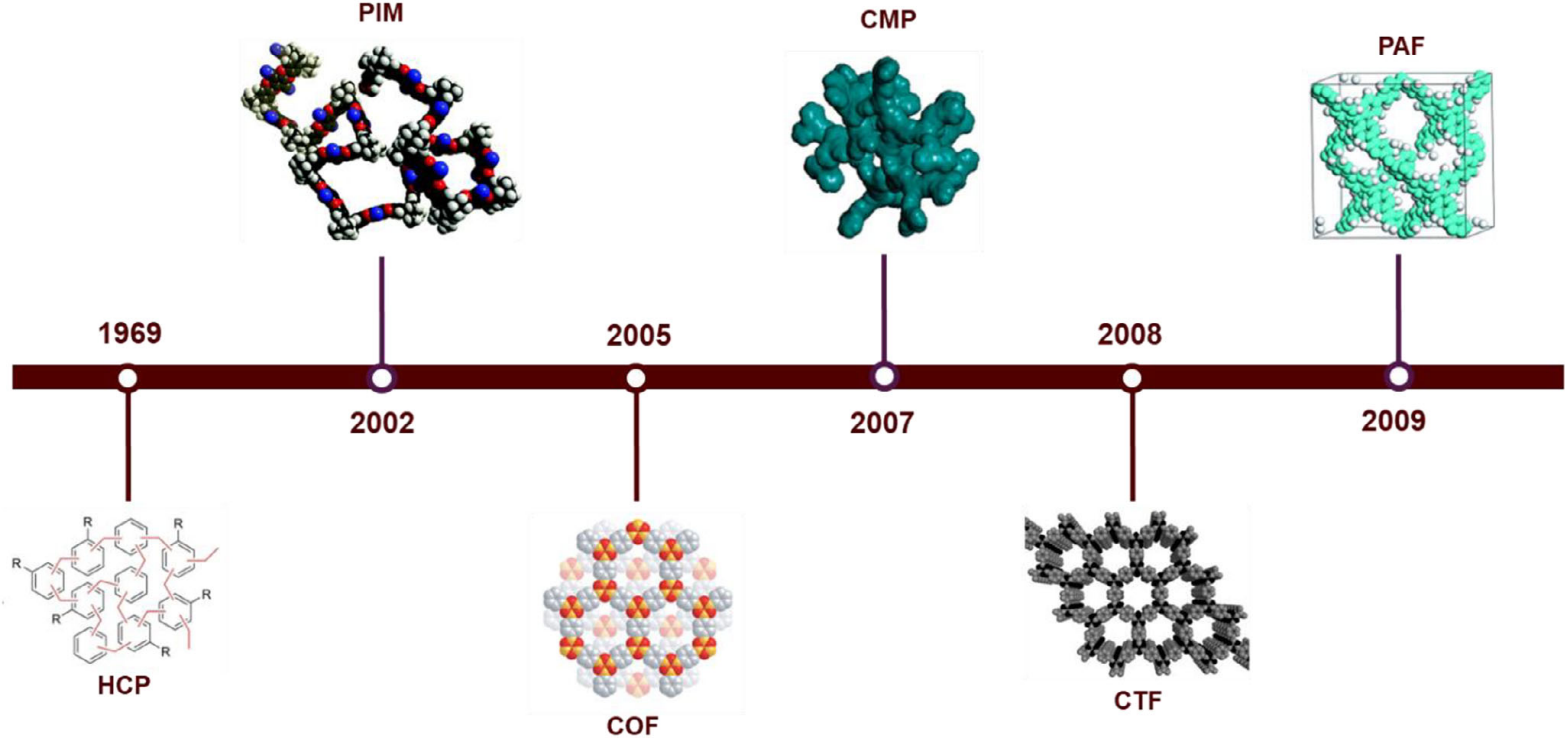
Illustration showing the sequential order of development of PPN. HCP structure was reproduced with permission [162]. Copyright 2024, Wiley‐VCH. PIM structure was reproduced with permission [146]. Copyright 2006, Royal Society of Chemistry. COF structure was reproduced with permission [163]. Copyright 2005, Science. CMP structure was reproduced with permission [164]. Copyright 2008, American Chemical Society. CTF structure was reproduced with permission [165]. Copyright 2008, Wiley‐VCH. PAF structure was reproduced with permission [166]. Copyright 2009, Wiley‐VCH.

HCPs are the oldest PPNs, formed by the crosslinking of polystyrene chains using Friedel–Crafts alkylation, and it was first reported by Davankov et al. in 1969 [167, 168]. Owing to their resin‐like character, they have been used in various applications like toxic chemical removal from solution, gas storage, and the stationary phase in chromatography. Pore engineering in HCPs, by varying the concentration of one constituent in the precursor, was first reported by Tan et al. in 2011 [169]. Recently, HCPs have again become popular due to the need for low‐cost materials for CO_2_ capture and catalytic conversion [170, 171, 172, 173]. On the other hand, McKeown et al. introduced PIMs in 2002, wherein they synthesized a phthalocyanine‐based PIM in which the phthalocyanine molecules were linked by a rigid spiro‐center that introduced intrinsic microporosity because of the orthogonal nature of the sorbent [174]. Due to their extensive surface areas and solubility in organic solvents, PIMs can be fabricated into membranes for practical applications [175]. While the HCPs and PIMs are amorphous, COFs are crystalline and were first reported by Omar Yaghi and co‐workers in 2005 [163]. The crystalline nature is ascribed to the reversible features of polymerization processes, which promote the synthesis of thermodynamically regulated polymers. This work was followed by the synthesis of 3‐D COFs using tetrahedral building blocks that bridged the gap between polymer science and crystallography [176]. To address the electronically insulating properties of PIMs and COFs, CMPs were constructed using p‐conjugated linkers, allowing for the electronic delocalization across the framework. Employing Sonogashira–Hagihara cross‐coupling reactions, Cooper and colleagues documented a sequence of CMPs: CMP‐1, CMP‐2, CMP‐3, and CMP‐4 [177]. In a key follow‐up study in 2008, Cooper showed that the pore size of CMPs can be controlled in a “quantized” fashion via statistical copolymerization of monomer struts with differing lengths [164]. Furthermore, CTFs formed by the cyclotrimerization of aromatic nitriles into triazine rings were first reported by Thomas and co‐workers in 2008 [165]. CTFs are semiconductive polymers, and their band gap has been tuned for application in photocatalysis [178]. PAFs are another class of PPNs with high surface area and ultra‐stability, due to their covalent bonding and rigid phenyl framework [179]. The first PAF synthesized by Yamamoto coupling was reported by Ben et al. in 2009, which possessed a diamondoid topology similar to the diamond lattice but expanded by phenyl rings [166]. This was adopted by Zhou and his team in 2010 to synthesize ultrahigh surface area materials that demonstrated excellent gas storage and separation [180].

## 6 Design and Application of Porous Polymer Networks in Light Hydrocarbon Separation

PPNs, as discussed in previous sections, have gained attention as a material for effective and efficient gas capture and separation [181, 182, 183, 184, 185, 186, 187, 188, 189]. This is because of their ultra‐high porosity, thermal, and chemical stability, in addition to the facile synthetic approach utilized in fabricating them from readily available monomers [189, 190, 191, 192]. They differ from sorbents such as MOFs [193, 194, 195, 196] and covalent organic frameworks (COFs) [197, 198, 199, 200, 201, 202] because they occur as amorphous sorbents due to the hyper‐crosslinking and irreversibility of their bonds, while the latter (MOFs and COFs) possess labile bonds that enable them to propagate as crystalline materials [157, 158, 159, 160, 203]. Although PPNs exist as amorphous materials, they still generate pores that are either microporous, mesoporous, or hierarchically porous for application in gas capture and separation/storage [204, 205, 206, 207]. Over the years, PPNs have been synthesized using Suzuki‐Miyaura [208, 209], Friedel–Crafts alkylation and acylation [210, 211], Yamamoto [207, 212], and oxidative coupling [213, 214] reaction route and applied in areas such as catalysis [158, 159, 184], energy storage [215, 216, 217, 218], and gas capture [156, 217, 219, 220, 221, 222, 223, 224, 225]. While gas capture applications have been explored, very little effort has been made in their design, development, and applications in light hydrocarbon separation. This is important because this class of sorbents has all the capabilities and higher advantages than others, such as MOFs and COFs, due to their higher stability, porosity, facile reaction procedure, readily available and cheap starting materials/monomers (Table 1). The hyper‐crosslinking and irreversibility of their bonds, and multiple random stacking, significantly contribute to their stability and application in harsh conditions. In this section, a detailed discussion of PPNs for light hydrocarbon separation will be explored, and potential candidates based on the structural properties of the reported sorbents (PPNs) will be suggested to boost attention to this aspect of sorbent research and development and drive alternative technology design for light hydrocarbon capture and separation to replace or modify the widely used cryogenic distillation technique that consumes tremendous amount of energy and cannot be sustained in the long term.

**TABLE 1:** Representative polymeric sorbents for light‐hydrocarbon purification and their reported adsorption/separation performance.

Sorbents (Refs.)	Structural features/BET surface area	Target separation	Uptake at stated conditions	Selectivity/Dynamic results	Conditions
PPN‐20 [226]	Graphene‐like porous network; robust in acidic, basic, and organic media	C_2_H_6_/C_2_H_4_; C_3_H_8_/C_2_H_6_/CH_4_	C_3_H_8_ 5.98; C_2_H_6_ 3.93; C_2_H_4_ 3.56; CH_4_ 0.8 mmol g^−^^1^	IAST C_2_H_6_/C_2_H_4_ = 2.20 (50/50); C_3_H_8_/C_2_H_6_ = 40.14 (50/50); C_2_H_6_/CH_4_ = 368.16 (50/50); C_3_H_8_/CH_4_ = 294 336.12 (50/50). Breakthrough produced >99% C_2_H_4_ and >99% CH_4_	298 K, 1 bar
PPN‐35 [124]	Hypercrosslinked, ultramicroporous (∼0.49 nm); 1637 m^2^ g^−^^1^	C_2_H_6_/C_2_H_4_; C_3_H_8_/C_2_H_6_/CH_4_	C_3_H_8_ 5.06; C_2_H_6_ 3.52; C_2_H_4_ 3.18; CH_4_ 0.79 mmol g^−^^1^	IAST C_2_H_6_/C_2_H_4_ = 1.67 (50/50); C_3_H_8_/C_2_H_6_ = 20.17 (50/50); C_2_H_6_/CH_4_ = 345.33 (50/50); C_3_H_8_/CH_4_ = 61907.55 (50/50). Breakthrough gave 99% C_2_H_4_ and 99% CH_4_	298 K, 1 bar
PAF‐1‐SO_3_Ag [15]	Ag(I)‐functionalized sulfonated PAF scaffold	C_2_H_4_/C_2_H_6_	C_2_H_4_ 4.1; C_2_H_6_ ∼2.2 mmol g^−^^1^	IAST C_2_H_4_/C_2_H_6_ = 27 (50/50); *Q*_st_(C_2_H_4_) = 106 kJ mol^−^^1^; >99% C_2_H_4_ in one cycle	296 K, 100 kPa
PAF‐109 [227]	Fluorinated microporous PAF	C_2_H_6_/CH_4_; C_3_H_8_/CH_4_; ternary C_3_H_8_/C_2_H_6_/CH_4_	C_3_H_8_ 1.93; C_2_H_6_ 1.23; CH_4_ 0.18 mmol g^−^^1^	IAST C_3_H_8_/CH_4_ = 556 (50/50); C_2_H_6_/CH_4_ = 22 (50/50)	298 K, 100 kPa
HCP‐B [228]	Pyridine‐triphenylborane HCP; ultramicroporous; 524 m^2^ g^−^^1^; stable to 400°C	C_2_ hydrocarbons/CH_4_	C_2_H_2_ 29.8; C_2_H_6_ 23.8; C_2_H_4_ 23.0; CH_4_ 9.5 cm^3^ g^−^^1^	IAST = 9.0 (C_2_H_6_/CH_4_), 8.1 (C_2_H_2_/CH_4_), 6.0 (C_2_H_4_/CH_4_), 50/50	298 K, 1 bar
P(Ph_4_Im‐Br‐DVB) [229]	Ionic porous polymer; stable in air for 6 months and to 280°C	C_3_H_4_/C_3_H_6_	C_3_H_4_ 56.2 cm^3^ g^−^^1^ at 273 k, 1 bar C_3_H_6_ 18.4 cm^3^ g^−^^1^ at 298 k, 1bar	IAST = 43 (1/99); >99.99% C_3_H_6_; capacity retained for >6 cycles	Mixture ratio 1/99 (v/v); temperature/pressure as reported in source study
Sorbents (Refs.)	Structural features/BET surface area	Target separation	Uptake at stated conditions	Selectivity/Dynamic results	Conditions
TT‐POP‐1/TT‐POP‐2 [230]	1,2,3‐Triazole‐functionalized triazine networks	C_2_H_4_/CH_4_; C_2_H_2_/CH_4_ (also CO_2_/CH_4_ and CO_2_/N_2_)	TT‐1: C_2_H_4_ 36.6, C_2_H_2_ 50.1 cm^3^ g^−^^1^; TT‐2: 19.8, 30.8 cm^3^ g^−^^1^	IAST TT‐1/TT‐2: C_2_H_4_/CH_4_ 14.7/13.6; C_2_H_2_/CH_4_ 40.1/48.4	298 K, 1 bar; 50/50
PAN‐AN/PAN‐NA/PAN‐P [14]	Ultramicroporous aromatic networks	C_2_H_6_/C_2_H_4_	C_2_H_6_: 2.38/3.45/3.00; C_2_H_4_: 1.97/3.02/2.68 mmol g^−^^1^	IAST = 1.68/1.53/1.28 (50/50); PAN‐AN retained integrity after 5 breakthrough cycles	298 K, 1 bar
APOP [34]	Anionic porous polymer; 428 m^2^ g^−^^1^; stable to 400°C	C_2_H_2_/C_2_H_4_; C_2_H_2_/C_2_H_6_	C_2_H_2_ 35.8; C_2_H_4_ 26.2; C_2_H_6_ ∼24.0 cm^3^ g^−^^1^	IAST = 1.9 (C_2_H_2_/C_2_H_4_) and 2.0 (C_2_H_2_/C_2_H_6_), 50/50; breakthrough validated	298 K, 1 bar
TPOA‐F/TPOA‐OH [231]	Fluoro‐/hydroxyl‐functionalized polyaminals; 1179/565 m^2^ g^−^^1^; stable >300°C	C_3_H_8_/CH_4_; C_2_H_6_/CH_4_; C_3_H_8_/C_2_H_6_	F: 4.25/3.09/∼0.75; OH: 2.13/1.30/∼0.33 mmol g^−^^1^ (C_3_H_8_/C_2_H_6_/CH_4_)	IAST F/OH: 99.0/136.5; 17.7/21.2; 7.4/5.7; unchanged after 5 cycles	298 K, 1 bar
CTF‐PO71 [232]	Pigment Orange 71‐derived CTF; hierarchical pores; 1401 m^2^ g^−^^1^	C_2_H_2_/C_2_H_4_	C_2_H_2_ 74; C_2_H_4_ ∼42.5 cm^3^ g^−^^1^	IAST = 1.8 (10/90) (C_2_H_2_/C_2_H_4_); breakthrough validated	298 K, 1 bar
FOSU‐POP‐1 [233]	Triazole‐containing click‐derived POP; 1038 m^2^ g^−^^1^	C_3_H_8_/CH_4_; C_2_H_6_/CH_4_; ternary C_3_H_8_/C_2_H_6_/CH_4_	C_3_H_8_ 2.85; C_2_H_6_ 2.14; CH_4_ ∼0.58 mmol g^−^^1^	IAST = 227 (5/95 C_3_H_8_/CH_4_) and 15 (10/90 C_2_H_6_/CH_4_); ternary breakthrough validated	298 K, 1 bar
P(Ph_3_Im‐Br‐0.5DVB) [234]	Rigid ionic imidazolium/bromide polymer	C_2_H_2_/CO_2_	C_2_H_2_ 34.3; CO_2_ 13.6 cm^3^ g^−^^1^	IAST = 17.9 C_2_H_2_/CO_2_ (50/50); *Q*_st_ = 40.9/29.0 kJ mol^−^^1^; stable over multiple breakthrough cycles	298 K, 1 bar

**Abbreviations**: BET, Brunauer–Emmett–Teller; CTF, covalent triazine framework; HCP, hypercrosslinked polymer; IAST, ideal adsorbed solution theory; PAF, porous aromatic framework; POP/PPN, porous organic/polymer network; *Q*_st_, isosteric heat of adsorption. Uptakes correspond to the conditions shown.

Festus et al. reported a series of four PPNs for light hydrocarbon separation [124]. The sorbents, which are PPN‐35, PPN‐45, PPN‐55, and PPN‐65, demonstrated capability for both binary (C_2_H_6_/C_2_H_4_) and ternary (C_3_H_8_/C_2_H_6_/CH_4_) gas mixtures separation. PPN‐35 had the highest IAST selectivity (1.67) for equimolar C_2_H_6_/C_2_H_4_ separation at 298 K and 1 bar and better properties (Figure 9). It also has a high BET surface area of 1637 m^2^/g and a C_3_H_8_, C_2_H_6_, C_2_H_4_, and CH_4_ adsorption of 5.06, 3.52, 3.18, and 0.79 mmol/g, respectively, at 298 K and 1 bar. Furthermore, it was able to separate a mixture of C_3_H_8_/C_2_H_6_/CH_4_ (30/30/40, v/v/v) to produce 99% pure CH_4,_ and equimolar C_2_H_6_/C_2_H_4_ (50/50) to produce 99% pure C_2_H_4_ as confirmed through a dynamic breakthrough experiment. PPN‐35's performance was reported to be because of ultra‐microporosity, which enabled molecular sieving of these gases and C–H···π interactions between the individual gas molecules and the PPN. This work is quite crucial in the development of PPNs for light hydrocarbon separation, as it shows that deliberate fabrication of PPNs with monomers that result in ultra‐micropores does not require further post‐synthetic modifications to either boost uptake or selectivity, thereby saving time and energy. In a follow‐up work, Festus, K. et al. reported the development of PPN‐20, which has the capability to purify C_2_H_4_ from a binary mixture of C_2_H_6_/C_2_H_4_ (50/50, v/v) and CH_4_ from a ternary mixture of C_3_H_8_/C_2_H_6_/CH_4_ (30/30/40, v/v/v), respectively [226]. This capability was validated through a dynamic breakthrough experiment. PPN‐20 produced >99% pure C_2_H_4_ and CH_4_ in the binary and ternary gas mixtures. This was because of the graphene‐like structure and pores that promoted the gas separation through a combination of C─H···π interactions, pore size matching, and van der Waals interactions. Due to the robustness of PPN‐20, which retained its structural integrity in both acidic, basic, and organic media, it is a viable candidate that can be up‐scaled and applied as an alternative to cryogenic distillation. Zhou and his team successfully fabricated PPN‐6‐SO_3_NH_4_ with a surface area of 593 m^2^/g for gas capture and separation [205]. The PPN‐6 scaffold acted as the basis for the anchoring of the ammonium sulfonate groups to improve selectivity in the gas adsorption process. After the anchoring of this functional group to the framework, the capability of PPN‐6‐SO_3_NH_4_ to adsorb CO_2_ was analyzed at 295 K and 1 bar. It was observed that the uptake, which was 0.9 mmol/g for the pristine framework, improved significantly to 3.4 mmol/g. This increase and change in the morphology of the pores lowered the methane adsorption capability. It had a Henry selectivity of 40 and an ideal adsorbed solution theory (IAST) of 180 for a 50/50 mixture of CO_2_/CH_4_. This high selectivity thus makes PPN‐6‐SO_3_NH_4_ a good candidate for CO_2_ purification from CH_4_. Additionally, due to the thermal stability, chemical properties, and pore size distribution, although ternary natural gas separation studies such as CO_2_/CH_4_/C_2_H_2_ were not conducted, this sorbent has the potential to purify them because of the presence of ultra‐micropores and polar pore environment. Shengqian Ma's group incorporated Ag (I) in the PPN‐6‐SO_3_H (PAF‐1‐SO_3_H) scaffold for the separation of C_2_H_4_/C_2_H_6_ [15]. Although C_2_H_4_ usually exists in small quantities in a C_2_H_4_/C_2_H_6_ mixture, preferentially adsorbing C_2_H_6_ over C_2_H_4_ is considered more energy efficient; the PAF‐1‐SO_3_Ag demonstrated significantly higher C_2_H_4_ selectivity (or adsorption capability) over C_2_H_6_ than any other reported PPN. While this does not follow the conventional expectation of C_2_H_6_ selectivity over C_2_H_4_, PAF‐1‐SO_3_Ag has a significantly high selectivity for C_2_H_4_. The structural stability of PAF‐1‐SO_3_Ag makes it a good candidate for this important binary gas purification. PAF‐1‐SO_3_Ag was observed to have an IAST of 125, an isosteric heat of adsorption (*Q*_st_) of 106 kJ/mol, and production of C_2_H_4_ >99% purity in a single cycle. It adsorbed 4.1 mmol/g of C_2_H_4_ and ∼2.2 mmol/g of C_2_H_6_ at 296 K and 1 bar. This unique capability of PAF‐1‐SO_3_Ag was experimentally determined to be a result of π‐complexation interactions of the Ag (I) in the framework with C_2_H_4_ by the *Q*_st_ calculations and in situ IR spectroscopy analysis. Wang et al. designed PAF‐109 for C_3_H_8_/CH_4_ and C_2_H_6_/CH_4_ purification [227]. This sorbent has a microporous pore size that facilitates the separation capability as observed from the IAST calculation based on the experimental data at 298 K and 1 bar. PAF‐109 has a C_3_H_8_, C_2_H_6_, and CH_4_ uptake of 1.93, 1.23, and 0.18 mmol/g at 298 K and 1 bar. It has an IAST selectivity of 22 for C_2_H_6_/CH_4_ (50/50) and 556 for C_3_H_8_/CH_4_ (50/50). The electronegativity of the fluorine atoms in the framework played a significant role in repelling the CH_4_ passage through the sorbent pores, which improved the selectivity. This was also validated through a successful dynamic breakthrough experiment for the separation of C_2_H_6_/CH_4_, C_3_H_8_/CH_4_, and C_3_H_8_/C_2_H_6_/CH_4_ (10/10/80). Chen et al. developed an HCP‐B, which is a hyper‐crosslinked porous polymer synthesized from pyridine‐triphenyl borane and Formaldehyde dimethyl acetal using the Friedel–Crafts alkylation reaction (Figure 10a) [228]. This PPN has high thermal stability (up to 400°C), which makes it unique, and also ultra‐micropores that boost its molecular sieving capability. Based on the N_2_ isotherm at 77 K and 1 bar (Figure 10b), it has a BET surface area of 524 m^2^/g and C_2_H_2_, C_2_H_6_, C_2_H_4_, and CH_4_ uptake of 29.8, 23.8, 23.0, and 9.5 cm^3^/g, respectively, at 298 K and 1 bar (Figure 10c,d). The variation in uptake of these light hydrocarbons is consistent with the molecular size or kinetic diameter of the molecule, which shows that the molecular sieving effect is also the driving force behind the selectivity, in addition to C‐H···π interactions between the framework and the gas molecules. HCP‐B has an equimolar IAST selectivity of 9.0, 8.1, and 6.0 for C_2_H_6_/CH_4_, C_2_H_2_/CH_4_, and C_2_H_4_/CH_4_ respectively, at 298 K and 1 bar. Based on the consistency of the IAST selectivity and variation in uptake of the various gases by kinetic diameter, it can be inferred that a breakthrough experiment, if performed, will be positive and show that this sorbent will be a good candidate to scaleup.

**FIGURE 9:**
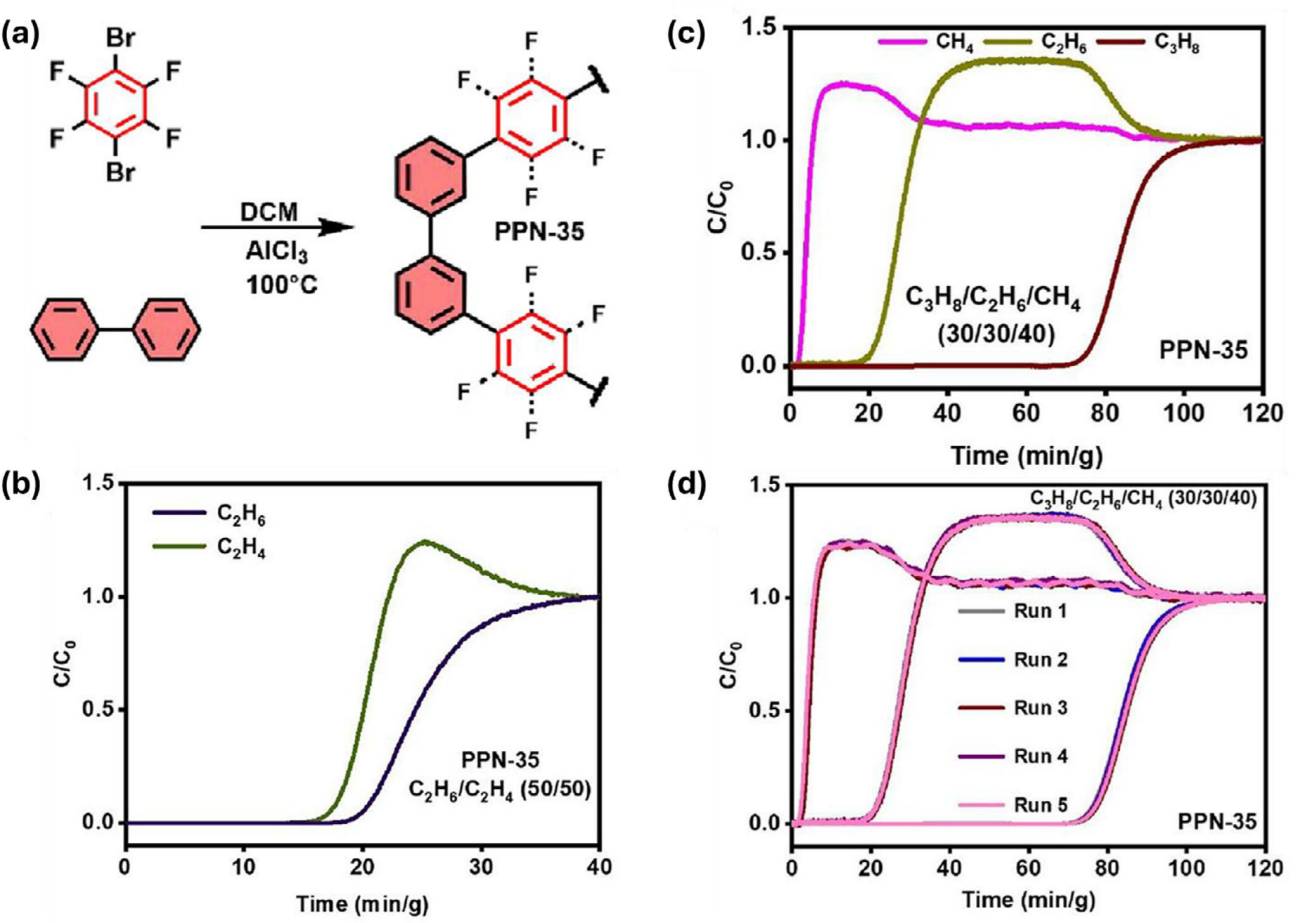
Synthetic scheme and properties of PPN‐35. (a) Synthetic scheme of PPN‐35 and the reaction conditions. (b) The equimolar binary breakthrough experiment of C_2_H_6_/C_2_H_4_ at 298 K and 1 bar. (c) The ternary dynamic breakthrough experiment of C_3_H_8_/C_2_H_6_/CH_4_ (30/30/40, v/v/v) at 298 K and 1 bar. (d) Multiple‐cycle ternary dynamic breakthrough experiment of C_3_H_8_/C_2_H_6_/CH_4_ (30/30/40, v/v/v) at 298 K and 1 bar. Reproduced with permission [124]. Copyright 2025, American Chemical Society.

**FIGURE 10:**
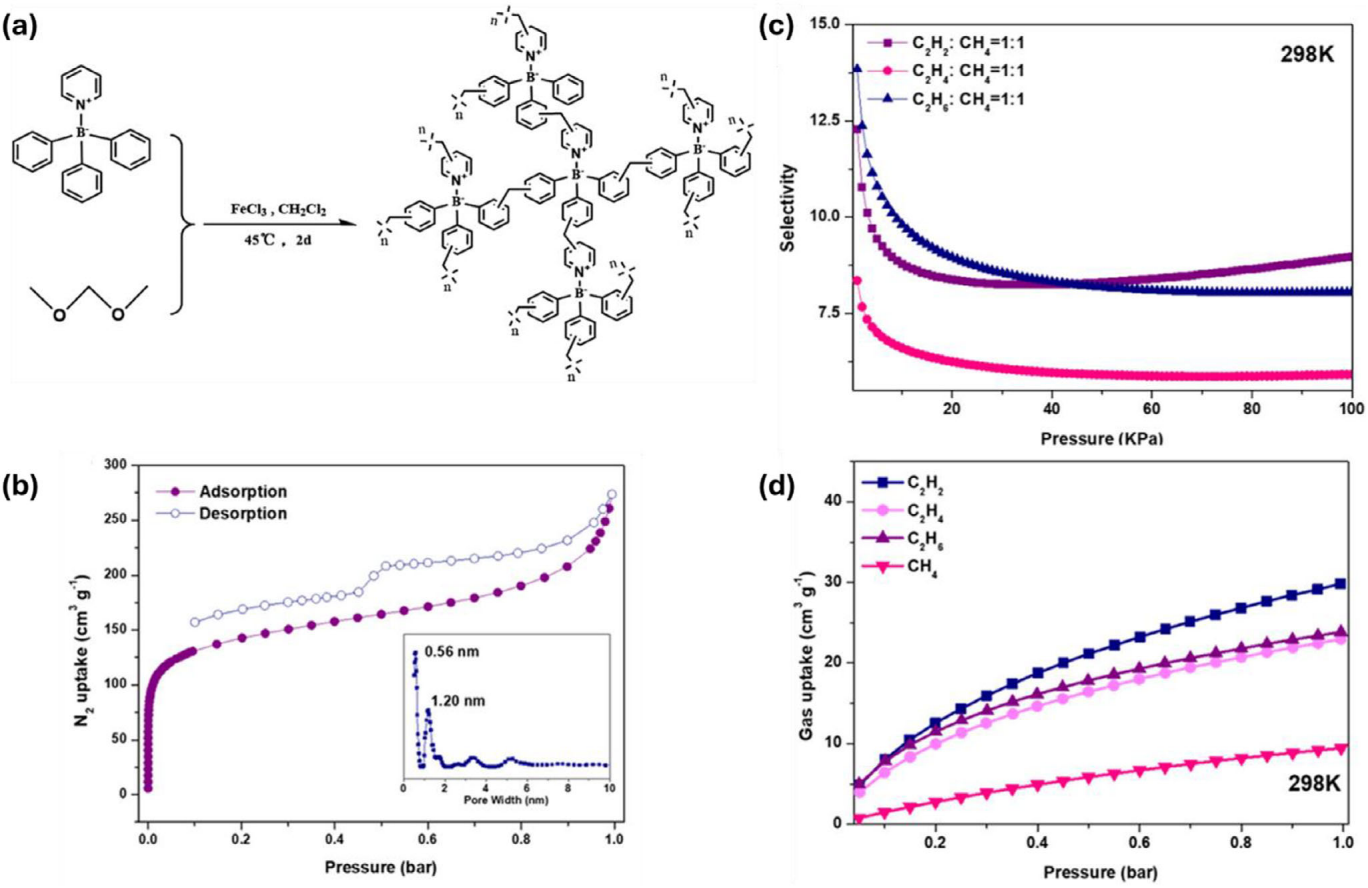
Scaffold and properties of HCP‐B. (a) Synthetic scheme of HCP‐B using Friedel–Craft alkylation. (b) Nitrogen adsorption isotherm of HCP‐B AT 77 K and bar. (c) C_2_H_2_, C_2_H_4_, C_2_H_6_, and CH_4_ adsorption isotherms at 298 K and 1 bar. (d) IAST selectivity of equimolar mixture of C_2_H_2_/CH_4_, C_2_H_4_/CH_4_, and C_2_H_6_/CH_4_ at 298 K and 1 bar. Reproduced with permission [228]. Copyright 2023, Wiley‐VCH.

Pan et al. developed P(Ph4Im‐Br‐DVB), which is an ionic PPN that could separate C_3_H_4_ from C_3_H_6_ to generate high‐purity C_3_H_6_ necessary for the manufacturing of polymer materials such as plastics [229]. This was achieved by taking advantage of the slight difference in their kinetic diameter to effectively separate them, which would be challenging with cryogenic distillation due to their extremely low boiling points. P(Ph4Im‐Br‐DVB) produced more than 99.99% pure C_3_H_6_ from C_3_H_4_/C_3_H_6_ mixture with no loss in its capacity after more than 6 cycles. It has an IAST of 43 for a C_3_H_4_/C_3_H_6_ (1/99, v/v) mixture, which is quite remarkable. Obviously, P(Ph4Im‐Br‐DVB) is a promising sturdy sorbent for application in the industry because, in addition to its separation capabilities, the scaffold remained stable after 6 months of exposure to air and demonstrated high thermal stability up to 280°C. In another work, a set of TT‐POPs (TT‐POP‐1 and TT‐POP‐2), PPNs which are actually 1,2,3‐triazole functionalized triazine‐based, were made by Liu et al. for CO_2_/N_2_, CO_2_/CH_4_, C_2_H_2_/CH_4_, and C_2_H_4_/CH_4_ separation [230]. At 298 K and 1 bar, the TT‐POP‐1 adsorption of CO_2_, C_2_H_4_, C_2_H_2_, and N_2_ was 30.6 cm^3^/g, 36.6 cm^3^/g, 50.1 cm^3^/g, and 2.9 cm^3^/g, while for TT‐POP‐2 it was 20.3 cm^3^/g, 19.8, 30.8, and 1.4 cm^3^/g, respectively. Although the selectivity of both sorbents does not consistently vary significantly for each binary gas tested, they both are useful for light hydrocarbon separation. This was validated by the IAST selectivity (50/50, v/v) of TT‐POP‐1 and TT‐POP‐2 for C_2_H_4_/CH_4_, C_2_H_2_/CH_4_, CO_2_/CH_4_, and CO_2_/N_2_ are 14.7, 40.1, 8.0, and 37.4, and 13.6, 48.4, 9.6, and 39.4, respectively. Overall, the uptake capability, IAST selectivity, and moderate *Q*_st_ show that a further breakthrough experiment will be positive, if conducted, and will validate the commercial usefulness of these sorbents in light hydrocarbon separation. Specifically for C_2_H_6_/C_2_H_4_, which is preferred to the reverse, C_2_H_4_/C_2_H_6_. Wang et al. successfully synthesized PAN‐AN, PAN‐NA, and PAN‐P, which demonstrated at 298 K and 1 bar, a C_2_H_6_ uptake of 2.38, 3.45, 3.00 mmol/g, and a C_2_H_4_ uptake of 1.97, 3.02, and 2.68 mmol/g [14]. Due to their ultra‐microporosity, PAN‐AN, PAN‐NA, and PAN‐P showed a 50/50 IAST selectivity of 1.68, 1.53, and 1.28 for a C_2_H_6_/C_2_H_4_ binary mixture at 1 bar and 298 K. The observed selectivities were because of the C‐H···π interactions between the framework and the C_2_H_6_ in addition to the ultra‐micropores present. The kinetic diameter of these gases, which are 0.44 and 0.42 nm for C_2_H_6_ and C_2_H_4,_ facilitated the molecular sieving effect. Obviously, PAN‐AN has the best performance of the three sorbents and retained its structural integrity after 5 breakthrough cycles. It shows that PAN‐AN is robust and promising for applications in the industry to generate ultrahigh‐purity ethylene from a C_2_H_6_/C_2_H_4_ mixture. Additionally, Jiang et al. used the Friedel‐Crafts alkylation reaction to develop an anionic porous polymer network, which was called (APOP), from potassium tetraphenylborate and dimethoxymethane for C_2_H_2_/C_2_H_4_ separation and purification [34]. This is important to produce C_2_H_4_ for polymerization processes and C_2_H_2_ as a source of energy. This sorbent has a surface area of 428 m^2^/g and high thermal stability up to 400°C. At 298 K and 1 bar, APOP had a C_2_H_2_, C_2_H_6_, and C_2_H_4_ uptake of 35.8, ∼24.0, and 26.2 cm^3^/g. The IAST selectivity was calculated to be 1.9 for an equimolar mixture of C_2_H_2_/C_2_H_4_. The breakthrough result supports the equimolar IAST for C_2_H_2_/C_2_H_4_ because the C_2_H_4_ breaks before C_2_H_2_. Further studies were also conducted for the purification of an equimolar mixture of C_2_H_2_/C_2_H_6_ with an IAST selectivity of 2.0 at 298 K and 1 bar. This validated that APOP can be used to purify C_2_H_2_ from a mixture of C_2_H_2_/C_2_H_4_ or C_2_H_2_/C_2_H_6,_ which is quite remarkable and useful. Two porous organic polyaminal frameworks (TPOAs), namely TPOA‐F and TPOA‐OH, were reported by Liu et al. (Figure 11a) [231]. The TPOA‐F was functionalized with fluorine, while the TPOA‐OH was functionalized with a hydroxyl group, and they both have a BET surface area of 1179 and 565 m^2^/g determined from the N_2_ isotherm (Figure 11b). The uptake of C_3_H_8_, C_2_H_6_, and CH_4_ was determined to be 4.25, 3.09, and ∼0.75 mmol/g for TPOA‐F and 2.13 mmol/g, 1.30 mmol/g, and ∼0.33 mmol/g for TPOA‐OH, respectively. Obviously the uptake follows the C_3_H_8_ > C_2_H_6_ > CH_4_ trend and IAST selectivity was 99.0, 17.7, and 7.4 for C_3_H_8_/CH_4_ (0.1/0.9, v/v), C_2_H_6_/CH_4_ (0.1/0.9, v/v), and C_3_H_8_/C_2_H_6_ (0.5/0.5, v/v) in TPOA‐F, and 136.5, 21.2, and 5.7 for C_3_H_8_/CH_4_ (0.1/0.9), C_2_H_6_/CH_4_ (0.1/0.9), and C_3_H_8_/C_2_H_6_ (0.5/0.5, v/v) in TPOA‐OH at 298 K and 1 bar. Both frameworks demonstrated high thermal stability above 300°C and didn't show any structure change after 5 regeneration cycles, which is essential for their advancement, upscaling, and commercialization. The presence of fluorine in TPOA‐F, hydroxyl groups in TPOA‐OH, and ultra‐micropores (Figure 11c) also contributed to the performance.

**FIGURE 11:**
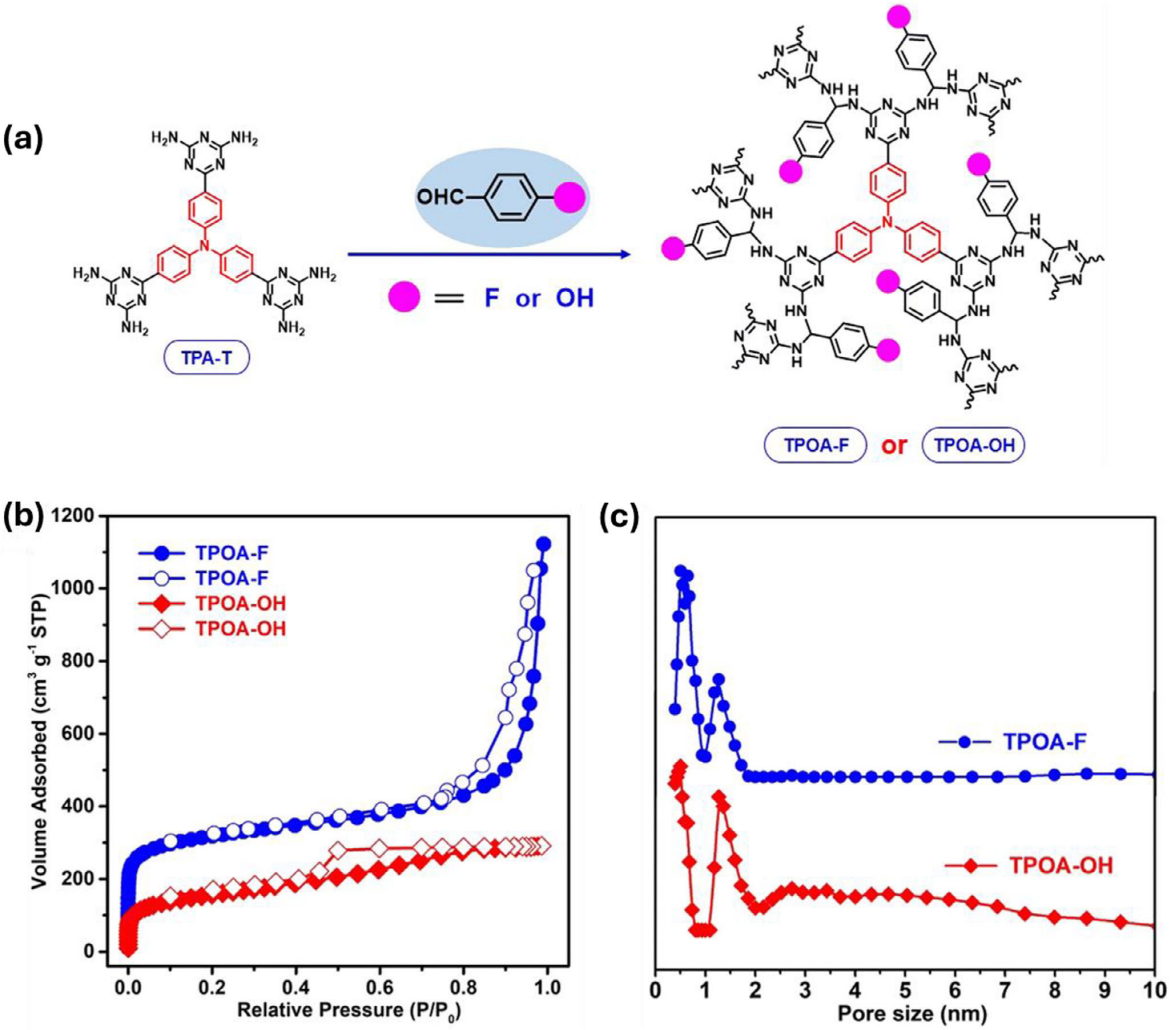
Synthesis and Properties of TPOA‐F and TPOA‐OH. (a) Synthetic scheme of TPOA‐F and TPOA‐OH. (b) TPOA‐F and Nitrogen sorption at 77 K and 1 bar. (c) Pore size distribution of TPOA‐F and TPOA‐OH at 77 K and 1 bar determined from the Nitrogen sorption Isotherm assuming a nonlocal density functional theory. Reproduced with permission [231]. Copyright 2023, Royal Society of Chemistry.

Lu et al. working on sorbents for the purification of C_2_H_2_ from C_2_H_4_, successfully developed CTF‐PO71, with pigment orange 71, which is a readily and commercially available monomer [232]. It has a BET surface area of 1401 m^2^/g. Due to the one‐step irreversible polymerization reaction, which likely causes interpenetration and random stacking, the sorbent was observed to have a high thermal stability and possessed hierarchical pores. At 298 K and 1 bar, it adsorbed 74 cm^3^/g of C_2_H_2_ and ∼42.5 cm^3^/g of C_2_H_4_. This difference in uptake shows that it has a selective adsorption of C_2_H_2_ over C_2_H_4_. Since the C_2_H_2_/C_2_H_4_ (10/90, v/v) IAST selectivity was 1.8 at 298 K and 1 bar, the practical selectivity capability was confirmed by a breakthrough experiment. Although CTF‐PO71 demonstrated outstanding performance in C_2_H_2_/C_2_H_4_ separation, its commercialization is not facile at this stage because of high temperature and long reaction time requirements for its fabrication. Modifying the synthetic route to become less energy and time‐consuming will facilitate its adoption in the light hydrocarbon separations in industrial processes. For ternary hydrocarbon gas separation, Wang et al. developed FOSU‐POP‐1 from the click reaction between tetrakis(4‐azidophenyl)methane and 1,3,5‐triethylbenzene [233]. FOSU‐POP‐1 has a surface area of 1038 m^2^/g and a C_3_H_8_, C_2_H_6_, and CH_4_ adsorption at 298 K and 1 bar of 2.85, 2.14, and ∼0.58 mmol/g. Before the ternary dynamic breakthrough experiment, the IAST selectivity at 298 K and 1 bar was determined to be 227 for C_3_H_8_/CH_4_ (5/95, v/v) and 15 for C_2_H_6_/CH_4_ (10/90, v/v). In a mixture ratio of 85/10/5 (v/v/v), the ternary breakthrough experiment of C_3_H_8_/C_2_H_6_/CH_4_ separation was done. It was observed from the breakthrough curve that the gases were separated based on their kinetic diameter. Since FOSU‐POP‐1 contains a triazole ring, the separation pattern is due to the high binding affinity of larger molecules (C_3_H_8_ and C_2_H_6_) than the smaller CH_4,_ together with the molecular sieving effect. Although this sorbent has good properties, its commercialization will be significantly difficult due to the high reaction temperature and time (220°C and 48 h.). Modifying the synthetic approach used will be a task of immense importance for the advancement of FOSU‐POP‐1 beyond bench scale and as a competitive alternative candidate for cryogenic distillation. Pan et al. focused on the separation of C_2_H_2_ from a C_2_H_2_/CO_2_ mixture and successfully achieved it with P(Ph3Im‐Br‐0.5DVB), an amorphous sorbent (Figure 12a) [234]. P(Ph3Im‐Br‐0.5DVB), an ionic polymer, has a C_2_H_2_ and CO_2_ uptake capacity of 34.3 and 13.6 cm^3^/g, respectively, at 1 bar and 298 K (Figure 12b). From the density functional theory (DFT) calculation, it was determined that this high IAST is because of the interaction between C_2_H_2_ and the bromide (Br–) ions. It has a *Q*_st_ of 40.9 kJ/mol and 29.0 kJ/mol for C_2_H_2_ and CO_2_, respectively, because C_2_H_2_ has a moderately high *Q*_st_ because of the strong interaction with the bromide ions anchored on the P(Ph3Im‐Br‐0.5DVB) framework (Figure 12c). Due to the rigidity of the structure, it retained its structural integrity after multiple breakthrough cycles of a mixture of C_2_H_2_/CO_2_ (50/50, v/v) at 298 K and 1 bar. This separation capability supported its IAST selectivity of 17.9 for a mixture of C_2_H_2_/CO_2_ (50/50, v/v) (Figure 12d).

**FIGURE 12:**
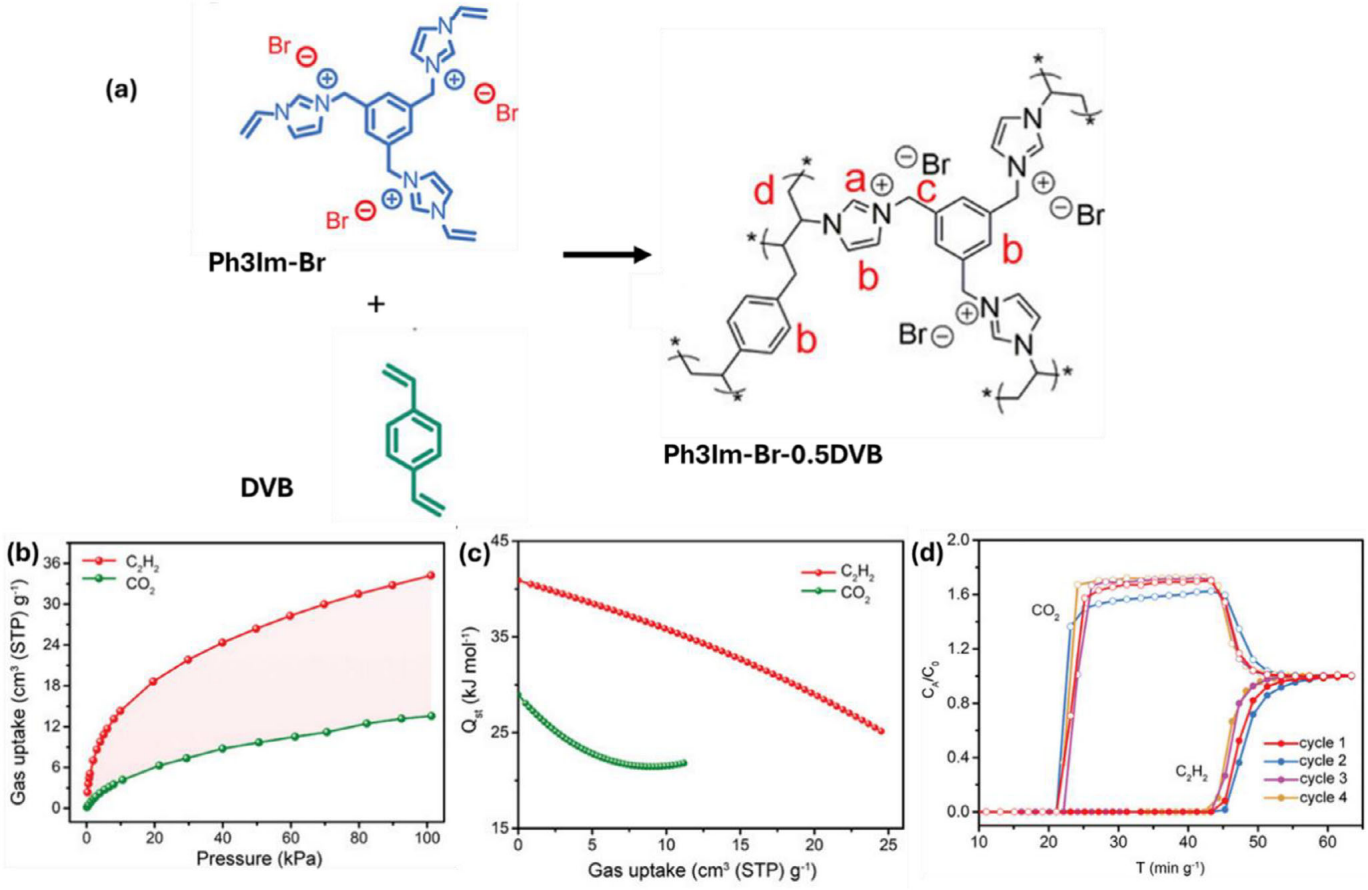
Synthetic Scheme and gas adsorption behavior of ionic porous polymer, P(Ph3Im‐Br‐0.5DVB). (a) The synthetic scheme of P(Ph3Im‐Br‐0.5DVB) with the bromine ion sites. (b) C_2_H_2_ uptake capabilities of P(Ph3Im‐Br‐0.5DVB) at 298 K and 1 bar. (c) Isosteric heat of adsorption of C_2_H_2_ and CO_2_ in P(Ph_3_Im‐Br‐0.5DVB) at 298 k and 1 bar. (d) The multiple breakthrough cycle of C_2_H_2_/CO_2_ equimolar binary gas mixture. Reproduced with permission [234]. Copyright 2023, Wiley‐VCH.

Furthermore, the separation of a C_2_H_2_, C_2_H_6_, and C_2_H_4_ ternary mixture, which is quite challenging because of the presence of two unsaturated hydrocarbons (C_2_H_2_ and C_2_H_4_), was achieved by Lee et al. [235]. In this work, Friedel‐Crafts reaction was used to synthesize a HCP through the solvent knitting method. HCP‐NO_2_‐1, a product of moderate nitration, reversed the separation trend from C_2_H_4_<C_2_H_6_<C_2_H_2_ using pristine HCP to C_2_H_4_<C_2_H_6_<C_2_H_2_. This is useful because it makes HCP‐NO_2_‐1 useful for both the separation of a ternary mixture of C_2_H_4_/C_2_H_6_/C_2_H_2_ and the binary mixture of C_2_H_4_/C_2_H_6_, respectively. This is fundamentally because of the electrostatic interaction between the nitro groups of HCP‐NO_2_‐1 with C_2_H_2_, thereby driving the preferential purification of C_2_H_6_ from C_2_H_4_ through C–H···π interactions. This concept is unique and seminal because it can be utilized in the development of PPNs for the purification of other gas mixtures that possess similar characteristics, thereby providing researchers with the post‐synthetic toolkit for engineering PPNs to achieve specific goals. It is important to note that in designing PPN for gas separation, especially in light hydrocarbon purification, success relies on certain mechanisms, which will be discussed in the next section. These mechanisms, if exhibited by the PPNs, will certainly give positive outcomes. The decision on the target gas mixture to be purified must be made before the fabrication of the PPNs. This is important because just like cryogenic distillation separates these gases based on temperature difference or gradient, PPNs (Table 1) perform the same task based on the mechanisms elaborated in the next section, at a much lower energy and instrument cost, with the ability to also withstand multiple separation cycles, harsh thermal and chemical conditions, and be up‐scalable.

## 7 Size Effect and Mechanism of Adsorption

The close similarity in molecular size, shape, and physicochemical properties of light hydrocarbons makes their separation particularly challenging using conventional cryogenic distillation. In recent years, PPNs have emerged as promising alternatives due to their structural tunability, chemical robustness, and hydrophobic pore environments. A central factor governing their separation performance is the size effect, which dictates adsorption selectivity through pore confinement, diffusion control, and specific host–guest interactions (Table 2).

**TABLE 2:** Structure–performance relationships and practical considerations for representative polymeric sorbents.

Sorbent (Refs.)	Dominant design/mechanism of adsorption	Practical advantages
PPN‐20 [226]	Graphene‐like π‐rich pores; pore‐size matching; van der Waals and C‐H···π interactions	>99% C_2_H_4_ and CH_4_ in breakthrough; strong chemical robustness
PPN‐35 [124]	Sub‐nanometer pore matching; molecular sieving; C‐H···π and dispersion interactions	High uptake; binary and ternary breakthrough; 99% product purity; no post‐synthetic functionalization
PAF‐1‐SO_3_Ag [15]	Ag(I)‐olefin π‐complexation	Very high C_2_H_4_ selectivity and single‐cycle >99% purity; structurally stable
PAF‐109 [227]	Fluorinated pore environment and size‐dependent adsorption suppress CH_4_	Very high C_3_H_8_/CH_4_ IAST selectivity; binary/ternary breakthrough demonstrated
HCP‐B [228]	Ultramicropore sieving plus C‐H···π interactions	Simple Friedel‐Crafts synthesis; high thermal stability; useful selectivity across several C_2_/CH_4_ pairs
P(Ph_4_Im‐Br‐DVB) [229]	Ionic pore sites and kinetic‐diameter discrimination	Ultrahigh‐purity C_3_H_6_; >6 cycles; long air stability and good thermal stability
TT‐POP‐1/TT‐POP‐2 [230]	Triazole‐rich polar pores increase affinity for C_2_ gases	Broad separation scope; moderate *Q*_st_ supports regeneration
PAN‐AN/PAN‐NA/PAN‐P [14]	Ultramicropore confinement and C‐H···π preference for C_2_H_6_	Ethane‐selective route enables direct high‐purity ethylene recovery; PAN‐AN stable for 5 breakthrough cycles
APOP [34]	Anionic pore environment plus differential adsorption of acetylene	One‐step Friedel‐Crafts route; high thermal stability; breakthrough confirmed for C_2_H_2_/C_2_H_4_
TPOA‐F/TPOA‐OH ([231])	Functional‐group polarity combined with ultramicropore confinement	High C_3_H_8_/CH_4_ and C_2_H_6_/CH_4_ selectivity; stable above 300°C and after 5 cycles
CTF‐PO71 ([232])	Hierarchical porosity and stronger C_2_H_2_ adsorption	High C_2_H_2_ capacity; commercially available monomer; breakthrough demonstrated
FOSU‐POP‐1 ([233])	Triazole binding sites plus size‐dependent molecular sieving	High C_3_H_8_/CH_4_ selectivity; ternary breakthrough demonstrated
P(Ph_3_Im‐Br‐0.5DVB) [234]	Br^−^···H‐C≡C interactions and imidazolium‐assisted binding	Strong C_2_H_2_/CO_2_ discrimination with moderate *Q*_st_; multiple breakthrough cycles

### 7.1 Molecular Size and Kinetic Diameter Effects

Short‐chain hydrocarbons (C_1_–C_3_) differ only subtly in size/shape, so separations often hinge on sub‐angstrom pore‐aperture matching and confinement [236, 237]. For instance, widely used kinetic diameters place CH_4_ near 3.8 Å and C_2_H_4_/C_2_H_6_ near ∼4.16/4.44 Å, while C_3_H_6_/C_3_H_8_ are typically around ∼4.5/4.3 Å. When PPN pore windows approach these dimensions (ultra‐micropores, <7 Å), molecular sieving becomes possible even for “difficult” pairs like C_2_H_4_/C_2_H_6_ (only ∼0.028 nm difference).

Although many high‐profile sieving demonstrations are in crystalline adsorbents (MOFs/zeolites), the same principle motivates PPN design, such as rigid backbones, narrow pore necks, and reduced segmental mobility, which can sharpen the effective pore aperture distribution and improve size discrimination [238, 239, 240, 241]. Recent work by Festus et al. provided compelling experimental validation that ultramicroporous PPNs can achieve size‐governed molecular sieving for C_1_–C_3_ hydrocarbons [239]. As discussed earlier, the systematic study of four hypercross‐linked PPNs (PPN‐35, PPN‐45, PPN‐55, and PPN‐65) with precise pore widths in the 0.49–0.64 nm range enabled highly selective separation of C_2_H_6_/C_2_H_4_ and C_3_H_8_/C_2_H_6_/CH_4_ mixture (Figure 13a). Among these materials, PPN‐35, with an ultramicropore width centered at ∼0.49 nm, exhibited the strongest molecular sieving effect, achieving a record equimolar C_2_H_6_/C_2_H_4_ IAST selectivity of 1.67 for a PPN, alongside exceptional equimolar IAST selectivities for C_2_H_6_/CH_4_, C_3_H_8_/C_2_H_6_, and C_3_H_8_/CH_4_ separations. Breakthrough experiments further confirmed that separation performance correlates directly with the kinetic diameter hierarchy (CH_4_ < C_2_H_6_ < C_3_H_8_) rather than solely adsorption enthalpy, with larger hydrocarbons experiencing delayed diffusion and longer residence time within the ultramicropores (Figure 13b). Importantly, adsorption capacities, uptake order, and *Q*_st_ followed molecular size and polarizability trends, reinforcing that pore confinement and steric restriction dominate selectivity over specific chemical binding effects in these systems. These findings establish ultramicropore engineering in PPNs as a viable route to molecular‐sieving‐driven hydrocarbon purification while retaining the robustness and moisture tolerance characteristic of polymeric networks.

FIGURE 13(a) Pore size distribution of PPN‐35, PPN‐45, PPN‐55, and PPN‐65 and their ultra‐microporosity. Reproduced with permission [239]. Copyright 2025, American Chemical Society. (b)Ternary breakthrough curves of C_3_H_8_/C_2_H_6_/CH_4_ (30/30/40, v/v/v) gas mixture for PPN‐35, PPN‐45, PPN‐55, and PPN‐65 at 298 K and 1 bar. Reproduced with permission [239]. Copyright 2025, American Chemical Society.

**Figure:**
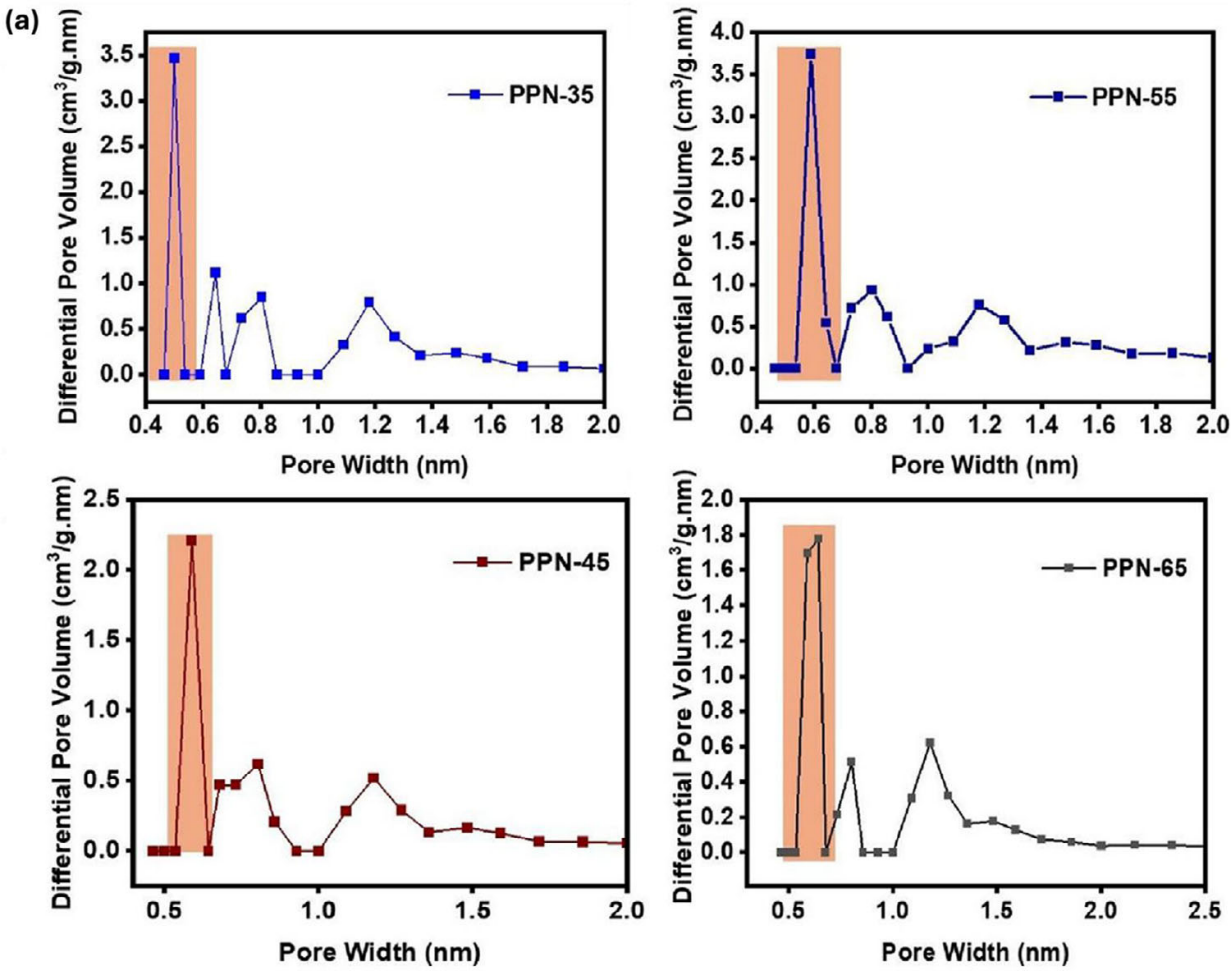

**Figure:**
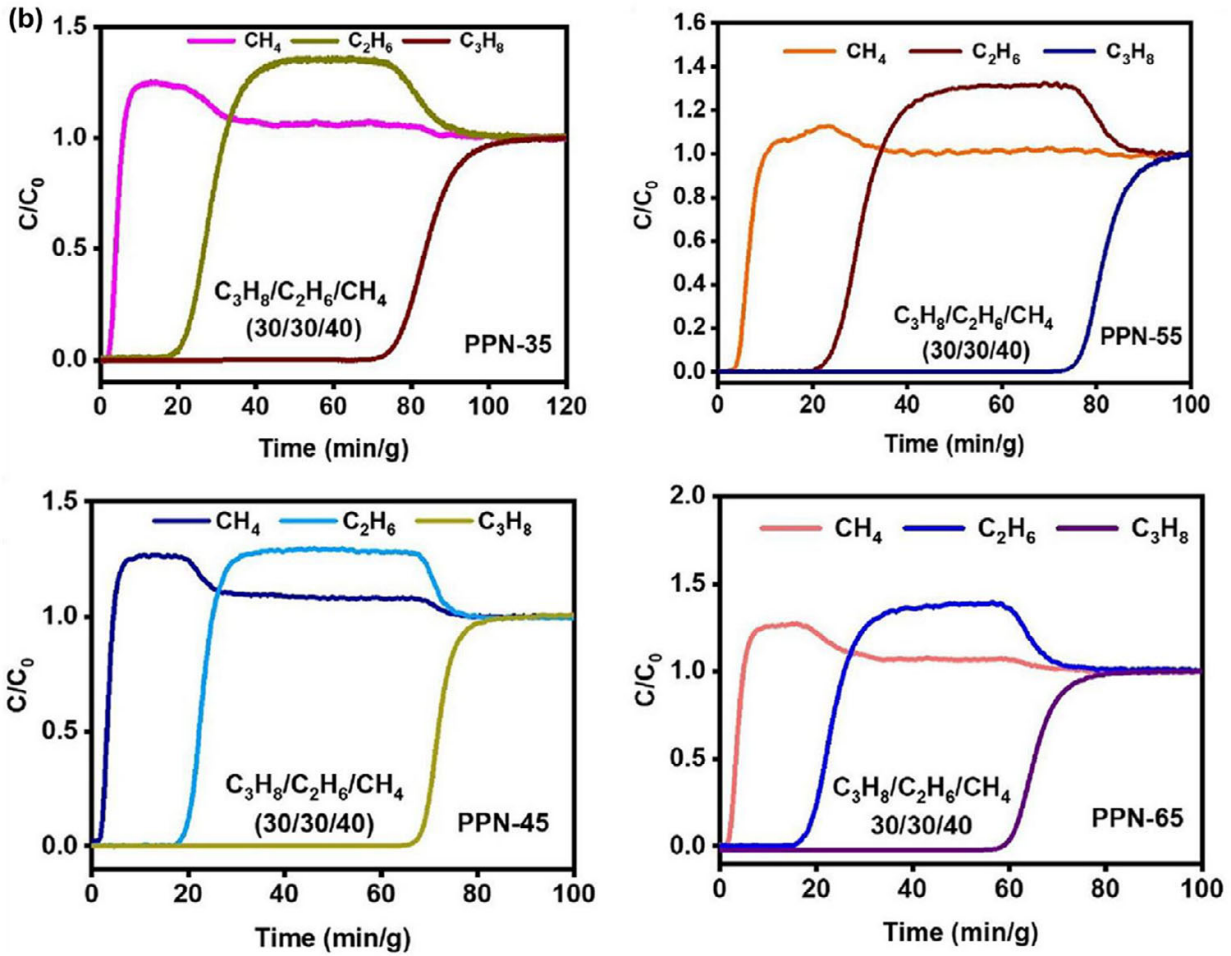

### 7.2 Adsorption Thermodynamics in PPNs

When steric exclusion is incomplete, selectivity becomes largely thermodynamic: larger/more polarizable hydrocarbons generally exhibit stronger dispersive interactions and higher adsorption enthalpies, while unsaturated hydrocarbons can gain additional stabilization through interactions with π‐rich pore walls or specific binding motifs [4, 31, 242, 243, 244, 245, 246, 247]. A well‐established strategy demonstrated prominently in PPNs, and related porous organics, is introducing π‐complexation (often via metal sites conceptually relevant as an “interaction design rule”) for preferential adsorption. Pan et al.'s work provides a representative example of an ionic Ph3Im‐Br, capable of highly selective C_2_H_2_/CO_2_ separation through tailored electrostatic and hydrogen‐bonding interactions rather than molecular sieving alone [234]. In this system, ultra‐microporosity ensures effective confinement, while high‐density bromide anions embedded within the pore channels act as specific binding sites for C_2_H_2_ via Br^─^···H─C≡C interactions.

DFT calculations revealed that C_2_H_2_ forms multiple cooperative interactions with the ionic framework, including hydrogen‐bonding and secondary C···H–N interactions with the imidazolium moieties, resulting in a significantly higher binding energy for C_2_H_2_ compared to CO_2_ (Figure 14a,b). Experimentally, this manifests as a substantially higher *Q*_st_ for C_2_H_2_ (∼41 kJ mol^−^^1^) than CO_2_ (∼29 kJ mol^−^^1^), confirming that enthalpic selectivity dominates the separation mechanism in this case. The moderate interaction strength enables facile regeneration, highlighting how balanced interaction energies rather than excessively strong binding are advantageous for practical separations.

**FIGURE 14:**
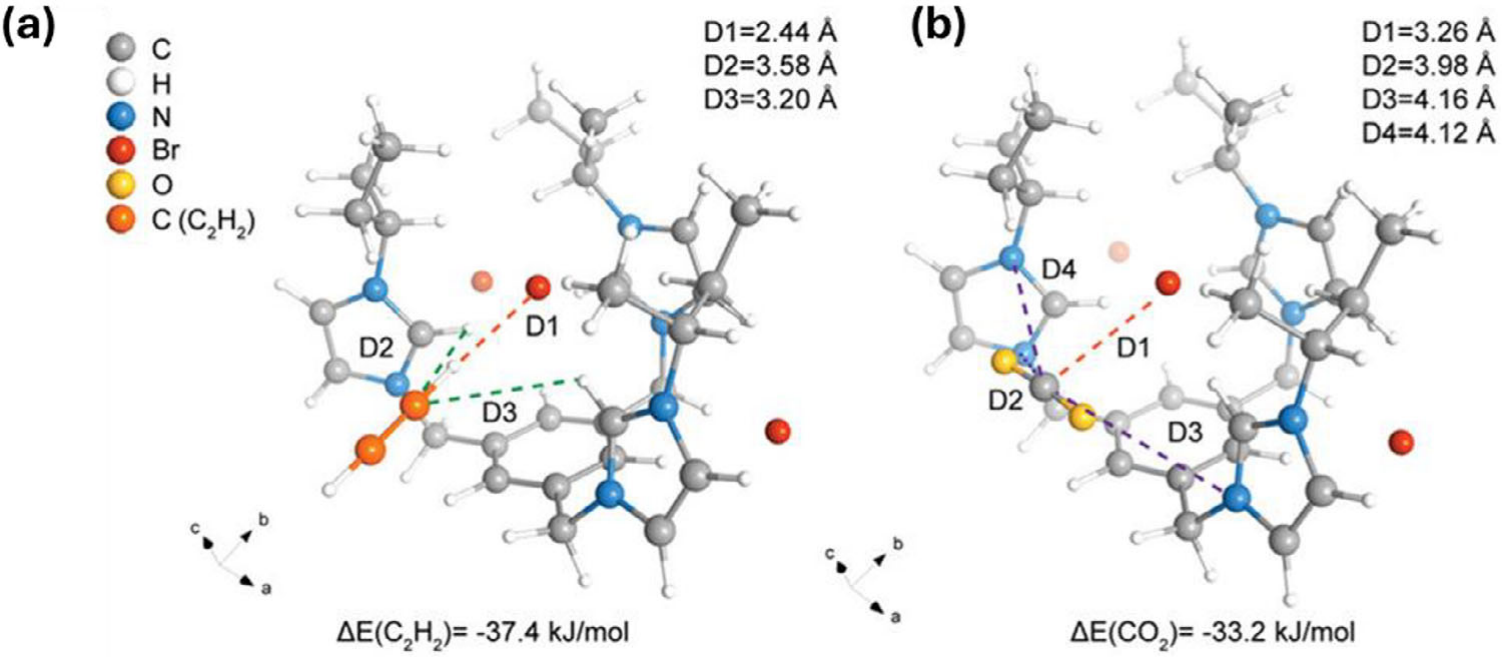
DFT‐based optimized geometries of the complexes with the interactions. (a) Ph3Im‐Br‐C_2_H_2_ and (b) Ph3Im‐Br‐CO_2_. Reproduced with permission [234]. Copyright 2023, Wiley‐VCH.

Zhou et al. demonstrated this concept through a family of functionalized porous aromatic frameworks (PAFs)‐PAF‐28, carbene‐functionalized cPAF‐28, and imidazolium‐functionalized iPAF‐28 designed specifically for C_2_H_2_/C_2_H_4_ separation [248]. *Q*_st_ calculations reveal that this selectivity originates from stronger host–guest interactions rather than molecular sieving, with C_2_H_2_ *Q*_st_ increasing from ∼26.7 kJ mol^−^^1^ in PAF‐28 to 35.0 kJ mol^−^^1^ in cPAF‐28 and 40.0 kJ mol^−^^1^ in iPAF‐28 (Figure 15a) while C_2_H_4_ binding remains comparatively weak. DFT analysis further shows that C_2_H_2_ preferentially interacts with the carbene carbon centers or undergoes synergistic binding with imidazolium rings and counter‐anions, forming multiple C–H···X interactions that stabilize C_2_H_2_ within the pore environment (Figure 15b). Dynamic breakthrough experiments corroborate these findings, confirming prolonged C_2_H_2_ retention and excellent recyclability under mixed‐gas conditions.

**FIGURE 15:**
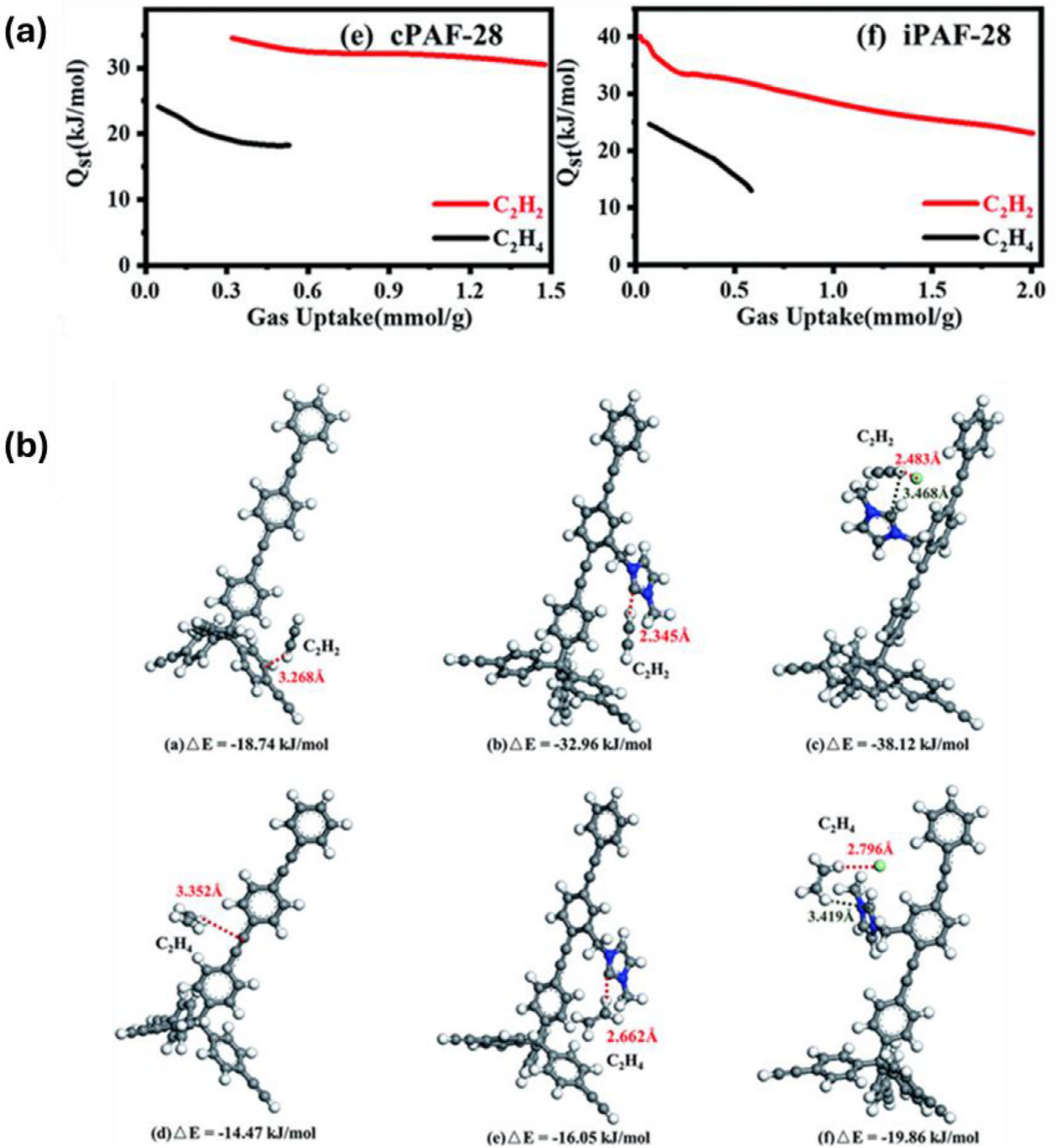
(a) Isosteric heats of adsorption (*Q*_st_) of C_2_H_2_ and C_2_H_4_ as a function of gas uptake for cPAF‐28 and iPAF‐28. (b) Preferred binding sites and calculated binding energies of PAF‐28 (a,d), cPAF‐28 (b,e), and iPAF‐28 (c,f) for C_2_H_2_ and C_2_H_4_. Reproduced with permission [248]. Copyright 2022, Royal Society of Chemistry.

### 7.3 Diffusion and Kinetic Selectivity

For close‐boiling hydrocarbon mixtures, kinetics can rival or dominate equilibrium. Narrow pore constrictions impose higher activation barriers for diffusion of bulkier guests; in size‐sieving or “bottlenecked” networks, this yields kinetic selectivity (fast adsorption/transport of one component; delayed uptake of another) [236]. This concept is widely developed in molecular‐sieving materials for C_2_/C_3_ separations and is directly obtainable in PPNs with tight ultra‐microporous windows.

Polymer‐based separations literature formalizes this trade‐off as diffusivity selectivity versus solubility (sorption) selectivity: larger, more condensable hydrocarbons tend to be more soluble/adsorbable, but smaller gases diffuse faster, especially in strongly size‐sieving matrices. For PPNs, the practical implication is that the observed selectivity (especially in breakthrough) can be controlled by (a) the tightest pore windows (governing diffusion) and (b) the strongest binding sites (governing thermodynamics), which may favor different species depending on chemistry and pressure/temperature (Table 2).

## 8 PPN Processing, Characterization, and Workflow

PPNs, owing to their insoluble and amorphous nature, are notoriously facile to characterize. Researchers employ a variety of methods, such as solid‐state CP/MAS ^13^C NMR, IR, SEM, TEM, SAXS, PXRD, and TGA. Solid‐state NMR, even though it has relatively low resolution, has been effective in providing molecular‐level information about PPNs [249]. Blanc et al. have developed an advanced technique called dynamic nuclear polarization (DNP) that dramatically improved the signal‐to‐noise ratios, enabling high‐throughput structural characterization of a library of porous polymers [250]. Hence, ^13^C NMR, hence, provides information on the aromatic, aliphatic, carbonyl, imine, and so forth, carbons present in the framework. Grätz and co‐workers demonstrated the characterization of porous polymers using DNP‐enhanced ^13^C and ^15^N ss‐NMR [251]. In this work, the mapping of 2D ss‐NMR was done to establish structural connectivity and network formation patterns. Just as important, FTIR also plays a significant role as a fast and commonly used technique for the characterization of functional groups in PPNs. It is capable of verifying the formation of new linkages like C─N, C─O, C≡N, C═N, in the polymer and the disappearance of precursor peaks present in the monomers (for example, ‐NH_2_, ‐B(OH)_2_, ‐Br, ‐Cl, ‐CHO, ‐COOMe, etc.) [252, 253, 254].

Due to the amorphous nature of PPNs and the lack of long‐range periodicity, further characterization of their morphology is done via SEM and TEM. SEM enables high‐resolution visualization of the porous polymer morphology (surface and cross section for roughness/thickness) and provides compositional/interfacial elemental distribution information through in situ EDX mapping [255, 256, 257, 258]. TEM is often used to investigate nanoscale morphology, particle size, and internal contrast related to porosity in PPNs. Gomes et al. reported the synthesis of a robust POP with high surface area (Figure 16a) characterized with FTIR (Figure 16b), HR‐TEM (Figure 16c), ^13^C CP MAS NMR (Figure 16d), UV–vis (Figure 16e), and FE‐SEM (Figure 16f). In this work, the TEM image revealed that the PPN has micropores of 1.3 nm dimensions, while it was observed from the SEM image that the interparticle distance corresponds to the average mesopores of 4.0 nm, which aligned well with the single‐component gas adsorption measurement [259].

**FIGURE 16:**
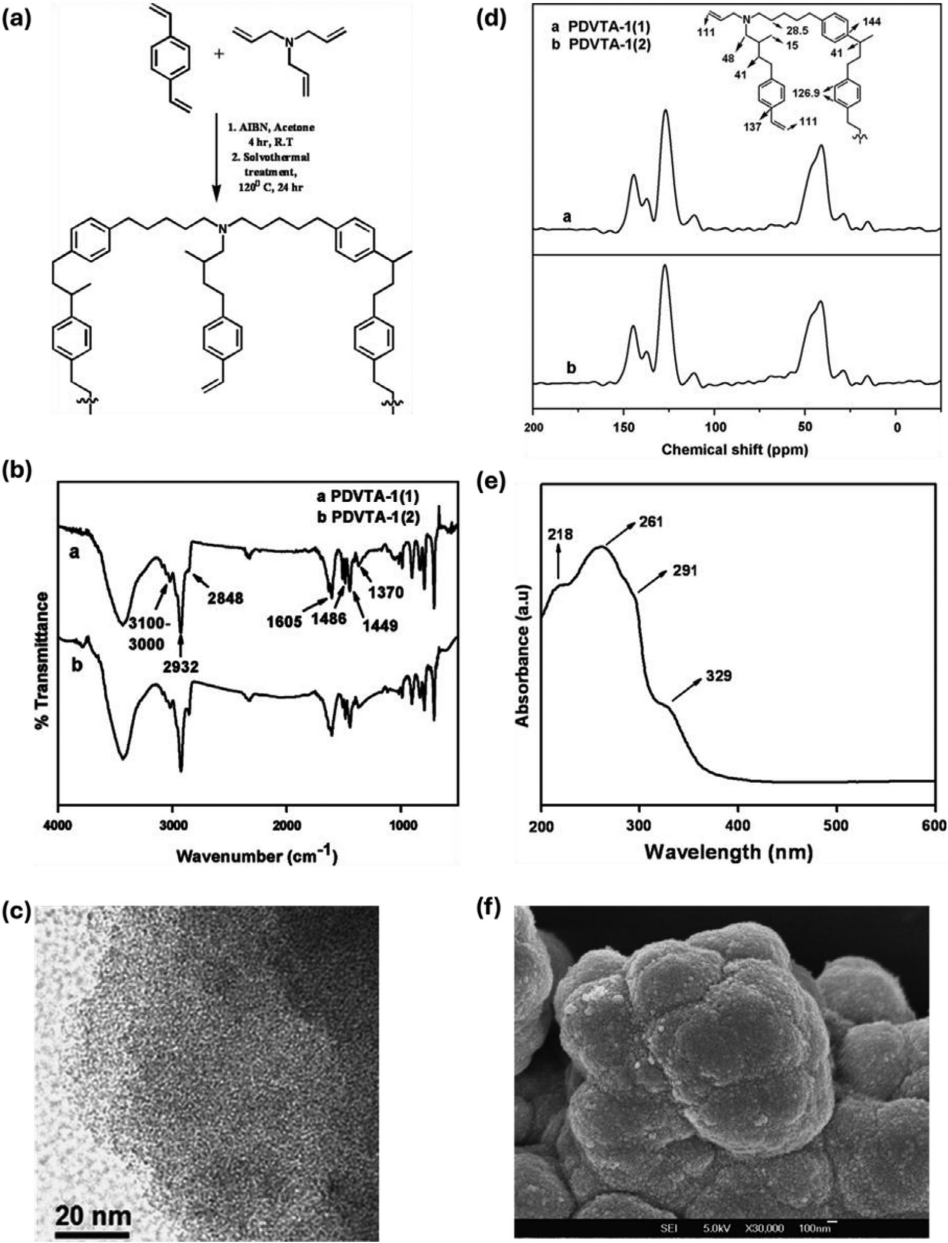
Synthetic scheme and characterization of PDVTA‐1 samples 1 and 2. (a) Synthetic reaction scheme. (b) FTIR spectra of PDVTA‐1 samples 1 and 2. (c) High‐resolution TEM of PDVTA‐1 structure. (d) ^13^C CP MAS NMR of PDVTA‐1 samples 1 (up) and 2 (down). (e) PDVTA‐1 sample 1 UV–vis spectrum. (f) Field emission SEM of PDVTA‐1. Reproduced with permission [259]. Copyright 2015, Elsevier.

Furthermore, PPNs, as mentioned earlier, are amorphous, that is, they lack ordered structures and crystallinity. Hence, their powder diffraction (PXRD) patterns are mostly broad peaks, but a few samples show observable microcrystalline domains at times [260, 261, 262, 263]. The thermal stability, decomposition behavior, and residual mass of these materials are determined using TGA [207, 264, 265]. Based on the workflow and processing of the material, TGA is performed before any gas adsorption test to determine the suitable activation temperature and to confirm the complete removal of solvents and guest molecules within the pores and on the surface. This is necessary to avoid random activation temperatures during gas uptake measurement that surpass the thermal stability of the material, as this certainly can lead to degradation or compromise of the sorbent's structural integrity.

For example, to assess selectivity and quantify hydrocarbon uptake, gravimetric and volumetric gas adsorption isotherms are used to calculate uptake ratios. IAST is a powerful predictive thermodynamic model used widely to estimate the selectivity of gaseous mixture uptake in porous sorbents [230, 266, 267]. Liu and co‐workers synthesized triazine‐based PPNs (TT‐POPs) and used IAST to calculate gas pair selectivities which were 40.1 for C_2_H_2_/CH_4_ and 14.7 for C_2_H_4_/CH_4_ using TT‐POP‐1; 48.4 for C_2_H_2_/CH_4_, 13.6 for C_2_H_4_/CH_4_ using TT‐POP‐2. Single‐site Langmuir–Freundlich (SSLF) fitting was used to derive IAST parameters with an excellent fit (R^2^> 0.999) [230]. The Langmuir–Freundlich (or Bradley‐Sips) model is the most widely used empirical equation for explaining adsorption data, especially for heterogeneous porous solids such as activated carbon and polymers [268].

Meng and co‐workers reported porphyrin‐based porous organic frameworks (POFs) named PAF‐40s, which showed high selectivity for C_2_ and C_3_ hydrocarbons over CH_4_ (Figure 17a) [269]. The thermal stability of the frameworks was ascertained with TGA (Figure 17b), while N_2_ adsorption at 77 K and 1 bar was done to determine the surface area and pore size distribution of the sorbents (Figure 17c). The uptake of the light hydrocarbon gases was measured, and their *Q*_st_ in each of the PAF‐40s was calculated by fitting the adsorption isotherm (Figure 17d,f) collected at 273 and 298 K to the virial equation. The reported *Q*_st_ ranged from 15.5–23.1 kJ mol^−1^ for CH_4_, 25.1–30 kJ mol^−1^ for C_2_H_4_, and 34.8–47.8 kJ mol^−1^ for C_2_H_6_, clearly indicating a trend of stronger interactions as the hydrocarbon chain length increases. Bracco et al. synthesized a series of high‐surface‐area, cross‐linked 3D polymers rich in phenyl and methylene linkages [270]. Based on the CH_4_ adsorption isotherm at 273 and 298 K, the *Q*_st_ was calculated, which ranged from 19–21 kJ mol^−1^. The gravimetric and volumetric isotherms of the materials revealed the intrinsic adsorption potential toward methane and carbon dioxide. To be specific, the gravimetric data showed appreciable per‐mass storage, while the volumetric measurements depicted the importance of packing efficiency and framework density for real‐life performance of these porous materials.

**FIGURE 17:**
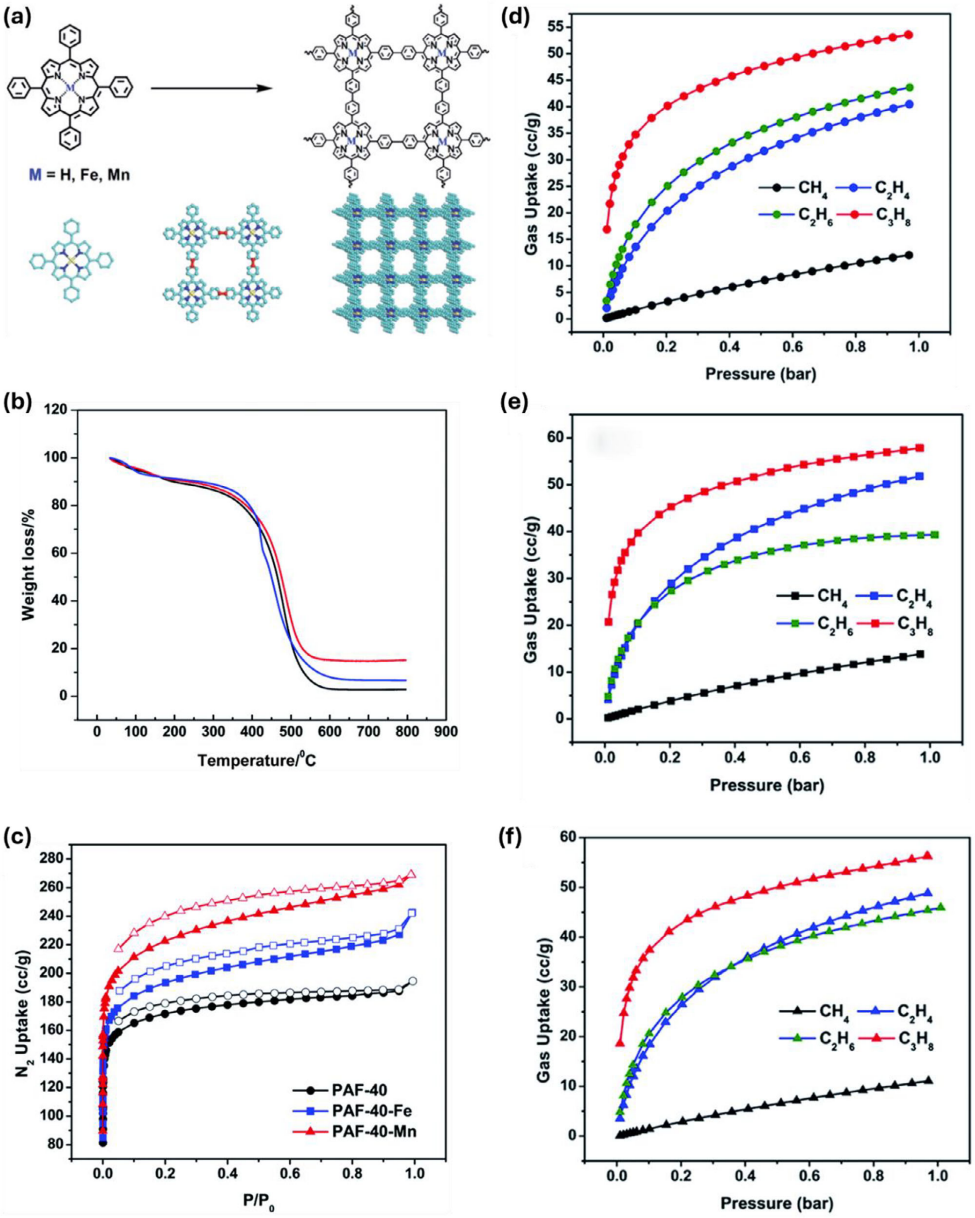
Synthetic scheme and characterization of PAF samples reported by Meng et al. (a) Synthetic scheme of PAF‐40s. (b) N_2_ uptake at 77 K and 1 bar. Gas uptake isotherms (CH_4_ black, C_2_H_4_ red, C_2_H_6_ blue, and C_3_H_8_ olive) for (c) PAF‐40, (d) PAF‐40‐Fe, and (e) PAF‐40‐Mn at 298 K and 1 bar. Reproduced with permission [269]. Copyright 2014 Royal Society of Chemistry.

Breakthrough analysis is an important experimental method in gas adsorption and separation research workflow. This is because it helps in bridging the gap between equilibrium adsorption data measured in labs and real industrial operating conditions. It is measured to evaluate how a particular adsorbate behaves under dynamic flow conditions; adsorption isotherms can be predicted directly from breakthrough data with only rough estimates of kinetic parameters, essentially eliminating the need for performing extensive equilibrium experiments [271]. Ma and co‐workers synthesized a family of porphyrinic ZnP‐CTFs through ionothermal trimerization using molten ZnCl_2_ (Figure 18) [272]. As part of the characterization of the sorbent, a breakthrough experiment was conducted to simulate the pressure‐swing adsorption (PSA) applicable in the industry. For the CH_4_/C_2_H_6_ mixture, CH_4_ eluted as a pure stream immediately, while C_2_H_6_ broke through after 17 min (CTF‐400) and 21 min (CTF‐500). For CH_4_/C_3_H_8_, CH_4_ was collected pure for 4–21 min (CTF‐400) and 5–25 min (CTF‐500), and for the ternary C_3_H_8_/C_2_H_6_/CH_4_ mixture, CH_4_ broke through first, C_2_H_6_ later, and C_3_H_8_ last, resulting in a clean separation of methane from C_2_–C_3_ fractions.

**FIGURE 18:**
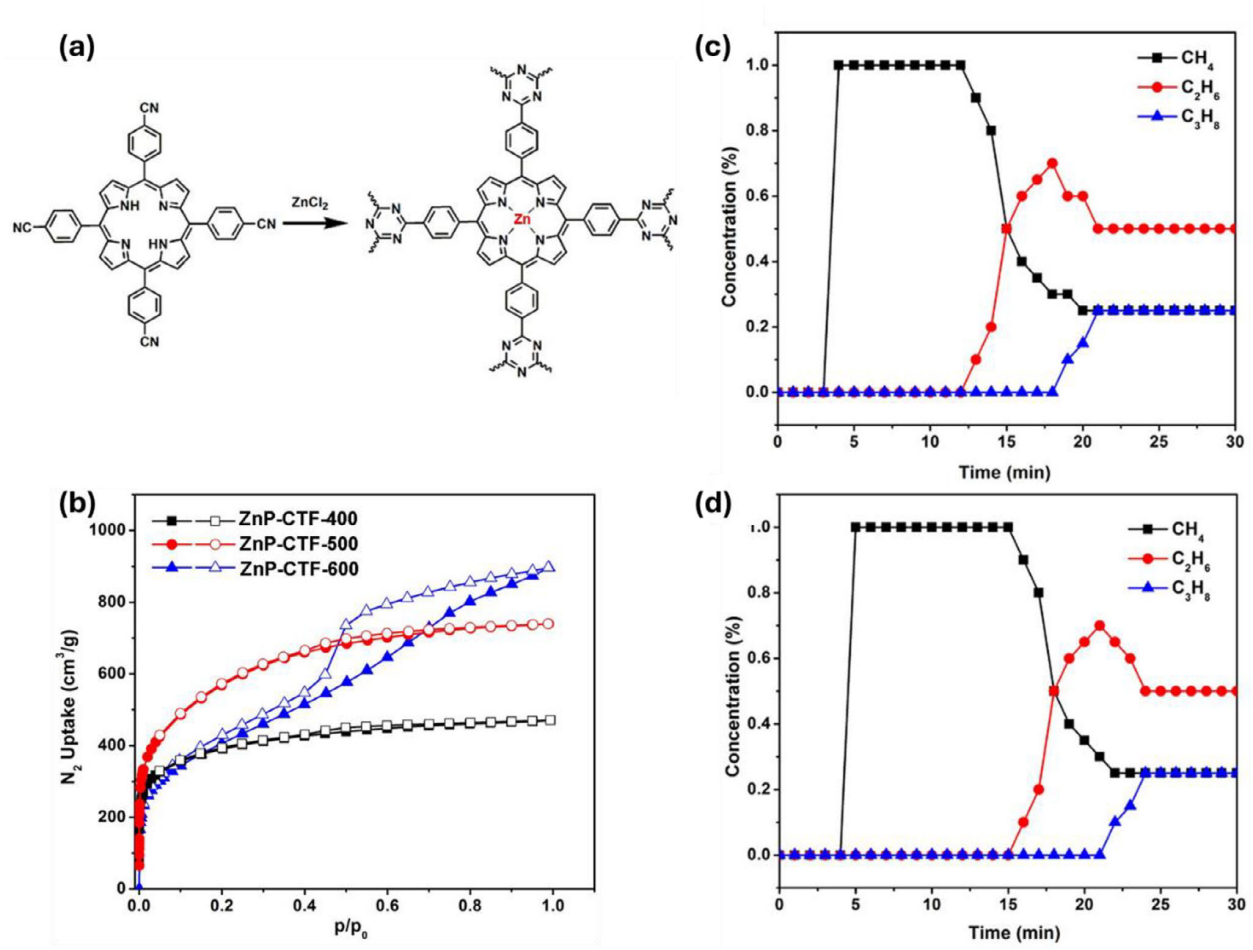
Synthetic schemes, gas adsorption, and breakthrough curves of ZnP‐CTF‐400, ZnP‐CTF‐500, and ZnP‐CTF‐600. (a) ZnP‐CTFs Synthetic scheme. (b) N_2_ isotherms at 77 K and 1 bar. (c) C_3_H_8_/C_2_H_6_/CH_4_ (25/50/25, v/v/v) breakthrough curve of ZnP‐CTF‐400 at 298 K and 3 MPa. (d) C_3_H_8_/C_2_H_6_/CH_4_ (25/50/25, v/v/v) breakthrough curve of ZnP‐CTF‐500 at 298 K and 3 MPa. Reproduced with permission [272]. Copyright 2013, Springer Nature.

Deng and co‐workers demonstrated real‐world separation metrics of their algae‐derived N‐doped porous carbon for a five‐component light hydrocarbon mixture (CH_4_, C_2_H_4_, C_2_H_6_, C_3_H_6_, C_3_H_8_) at 298 K and 1 bar [273]. The breakthrough sequence was as follows: C_3_H_8_ (76.5 min) ≈ C_3_H_6_ (76.2 min) > C_2_H_6_ (33.7 min) ≈ C_2_H_4_ (30.6 min) > CH_4_ (11.6 min). Hence, it was possible to obtain a pure CH_4_ stream, owing to the longer retention of heavier hydrocarbons. These tests validated that ANPC‐2‐700 can separate a multicomponent light hydrocarbon mixture into three high‐purity grade fractions (C_1_, C_2_, and C_3_). There are fewer reports of breakthrough results of PPNs as compared to materials like MOFs, zeolites, and other carbon‐derived materials [123, 274, 275, 276, 277, 278, 279]. The workflow and characterization techniques enumerated in this section will be instrumental in facilitating the fabrication of PPNs by providing the road map needed and, hence, eliminating any roadblocks. In summary, the traditional workflow is thus: (a) synthesis, (b) thermal stability test, (c) sample activation, (d) gas uptake measurements, (e) selectivity and heat of adsorption calculations, (f) structure, functional group, and morphology determination, (g) dynamic breakthrough analysis, and (h) any other supporting miscellaneous studies (which sometimes are recommended but not required).

In general, gas adsorption isotherms (gravimetric/volumetric) provide direct measurements of adsorption performance, including uptake at specified temperatures and pressures, working/deliverable capacity, and, sometimes, kinetic data [180, 207, 280, 281, 282, 283]. For instance, a series of triptycene‐based amine‐linked POPs were synthesized by Alam et al. [283]. The CH_4_ uptake was measured by volumetric adsorption experiments at 273 and 298 K at 1 bar. The CH_4_ adsorbed via physisorption was measured in mg/g as a function of applied pressure (P/P_0_). All the adsorption isotherms were reversible, confirming physisorption. Jiang et al. synthesized a conjugated microporous polycarbazole network showing CH_4_ storage capability [284]. A static volumetric gas adsorption isotherm (physisorption) was studied at 273 and 298 K at 1.1 bar. The sorbent showed strong potential for CO_2_/CH_4_ separation because of its ultra‐microporosity and nitrogen‐rich sites. From these absorption studies, the *Q*_st_ were calculated using the Clausius‐Clapeyron equation. The higher the difference in *Q*_st_ between two adsorbed gases, the higher the expected selectivity. This further makes it obvious that (a) ultra‐microporosity, (b) selectivity, and (c) isosteric heat of adsorption are important parameters that must be considered before the design or fabrication of any PPN for optimal performance.

## 9 Conclusion and Outlook

Traditionally, cryogenic distillation has been at the core of light hydrocarbon purification. It has been highly reliable and capable of achieving the ultrahigh purity necessary for the production of polymers and sustainable energy. Nevertheless, the process has been associated with considerable energy costs, which necessitate an alternative. It operates at very low temperatures, which are thermodynamically expensive and not sustainable due to rising energy costs. Although some improvements have been realized through process optimization techniques, this has not significantly reduced the energy consumption of the process.

In light of the above, the emergence of PPNs has been highly positive and credible. This is because their chemical and thermal stability are highly desirable in light hydrocarbon purification. Furthermore, the ability to modify their pore structure, distribution, and environment is highly beneficial to the development of superior sustainable purification technology. Although the amorphous characteristics of PPNs were previously viewed as a disadvantage, this property is currently viewed as essential because of the interpenetration and random stacking of the layers during polymerization, which improves their thermal, chemical, and mechanical capabilities. Recent advances have clearly demonstrated that without the need for long‐range crystallinity, PPNs are able to exhibit significant selectivity via ultra‐micropore confinement, host‐guest chemistry, and diffusion‐based separation mechanisms.

On the basis of the literature that has been reported to date, several design strategies have come to the forefront as being of particular importance in the development of high‐performance PPNs for gas separations. These include the need for highly precise control over pore size at the sub‐nanometer level in order to take advantage of the very small differences in kinetic diameter between closely related hydrocarbon molecules for effective separation. There must be a balance between the strength of adsorption, as this must be strong enough to provide a reasonable degree of selectivity, but not so strong that regeneration becomes inefficient or energy demanding. Dynamic breakthrough experiments have been found to be of crucial importance in the evaluation of separation performance, since equilibrium selectivity does not necessarily correlate with efficient separations under realistic operating conditions.

Despite the advantages of these PPNs, there are still challenges to overcome to facilitate their adoption, integration into modern light hydrocarbon purification technology, and commercialization. The inability to perfectly control the pore size distribution in PPNs due to their amorphous nature seems to be the most important. To this effect, some researchers have established the choice of starting materials (monomers) to be essential in predicting and achieving specific pore sizes and distributions, but this stills further investigations and optimizations. Furthermore, the integration of modelling, characterization, and monomer design must be increased to an even higher level than it is currently to help clarify the relationships between the structure and properties of PPNs.

Finally, although the process of cryogenic distillation is widely used in the industry, adsorption‐based alternative, however, provide a viable means of mitigating the energy demands of light hydrocarbon purification. Rather than replacing the process of cryogenic distillation, PPNs might play roles in hybrid forms of the process soon before it will completely replace cryogenic distillation in the near future. The rapid expansion of PPN research for light hydrocarbon separation indicates a growing interest in adsorbent‐based materials for gas purification. As more researchers from fundamental and applied sciences join in advancing PPNs, this will play an important role in the development of more sustainable hydrocarbon purification technologies.

## Conflicts of Interest

The authors declare no conflicts of interest.

## Data Availability

Data sharing not applicable to this article as no datasets were generated.
